# Tere Tohorā, Karanga Tāngata: Weaving Māori Knowledge With Conventional Science to Characterise a Biodiversity Hotspot for Marine Megafauna in an Area Facing Multiple Anthropogenic Impacts

**DOI:** 10.1002/ece3.72558

**Published:** 2025-12-15

**Authors:** Tom Brough, Hollie Kereopa, Taryn Shirkey, Jochen Zaeschmar, Eva Leunissen, Dave Milner, Juliane Chetham

**Affiliations:** ^1^ Earth Sciences New Zealand Dunedin New Zealand; ^2^ Patuharakeke Te Iwi Trust New Zealand; ^3^ Far Out Ocean Research Collective Charitable Trust Pahia New Zealand

**Keywords:** dual knowledge systems, marine mammal, marine megafauna, mātauranga Māori, seabird, traditional ecological knowledge

## Abstract

Marine megafauna are important components of marine ecosystems and are of major significance to Indigenous communities, including Māori. Despite being recognised as a biodiversity hotspot for megafauna, most locations in Aotearoa New Zealand (NZ) do not have adequate information for the management of anthropogenic impacts on these taxa. Due to long‐standing relationships with Māori, there is a wealth of mātauranga Māori (Māori knowledge) on megafauna that may help fill key gaps. This study, Tere Tohorā Karanga Tāngata, aims to address information gaps on marine megafauna within Te Ākau/Bream Bay, on the north‐east of NZ's North Island. We utilise a wānanga (shared learning) approach alongside conventional visual and acoustic surveys to synthesise an accurate baseline of species occurrence, distribution, habitat use and site fidelity. Māori knowledge in a variety of forms was gathered and simultaneously integrated into the survey design across seven vessel‐based field surveys. We calculate seasonal sighting rates and use species distribution models (SDM) to determine the distribution of commonly occurring species and use photo‐identification to investigate site fidelity of marine mammals. Both knowledge systems confirmed the importance of Te Ākau/Bream Bay for marine megafauna, reporting high diversity and abundance of marine mammal (8 species) and seabird species (24 species) and high sighting rates of threatened taxa. While most species were encountered year‐round, sighting rates and predictions from SDMs highlight seasonal variability in the occurrence and distribution of most species. Māori knowledge aligned closely with results from conventional scientific approaches in confirming the most common species (common dolphin, Bryde's whale), seasonal habitat preferences, and, importantly, provided historical information on species that have been extirpated from the study area. Combined, the two knowledge systems have generated a robust baseline on marine megafauna that can be used to guide the management of these important species and showcase the advantages of utilising dual‐knowledge systems for characterising marine biodiversity.

## Graphical abstract

This study weaves traditional Māori knowledge and conventional scientific surveys to generate a stocktake on the biodiversity of marine megafauna in an area facing multiple anthropogenic impacts in New Zealand. Across dual knowledge systems, we show that Te Ākau/Bream Bay provides critically important habitat for a range of marine mammal and seabird species. Our study demonstrates the significant value of considering traditional ecological knowledge when generating baseline studies of marine megafauna.

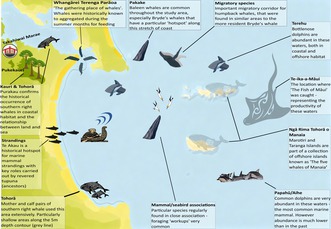

## 1 Introduction

In an era of significant impacts of human activity on marine biodiversity, obtaining appropriate baseline data on the status of key species and habitats is of critical importance. This information can guide sustainable management of the marine environment and enable robust monitoring programmes to identify and mitigate adverse impacts (Lotze and Worm 2009; Borja et al. 2012). The value of including Indigenous Knowledge (IK) (also referred to as Traditional Ecological Knowledge) in baseline assessments has been recognised across a range of marine systems, with benefits accruing due to the ability to draw evidence from multiple ‘ways of knowing’ (Gagnon and Berteaux 2009; Thornton and Scheer 2012; Teixeira et al. 2013; Alexander et al. 2019; Moore and Hauser 2019; Stern and Humphries 2022; Gryba et al. 2025). Interweaving of IK with conventional science can be undertaken in several ways, using both quantitative and qualitative methodologies that incorporate IK into study design, analysis of species‐habitat relationships, distribution and population modelling, and general information on species ecology, among others (Gagnon and Berteaux 2009; Alexander et al. 2019; Stern and Humphries 2022; Gryba et al. 2025). However, inclusion of such knowledge in baseline assessments is not widely adopted. Mismatches in the spatial and temporal scales of both historical and contemporary experiential information that often forms the basis of IK and Western science can result in contrasting information (Alexander et al. 2019; Stern and Humphries 2022), yet this mismatch can in itself expand information on species ecology (Gagnon and Berteaux 2009). Additionally, ineffective communication, lack of participation/involvement in research, and the requirement for trust‐based relationships among practitioners from both backgrounds can limit effective interweaving of knowledge (Thornton and Scheer 2012; Ataria et al. 2018; Moore and Hauser 2019; Marsh et al. 2022; Stern and Humphries 2022).

Marine megafauna (marine mammals, seabirds, reptiles, large fish and sharks) are a key component of Aotearoa New Zealand's (hereafter NZ) marine ecosystems and are of substantial importance to Māori *iwi/hapū* (tribes/sub‐tribes) and other coastal communities (Haami 2012; Pryor Rodgers 2017; Stewart 2024). Megafauna are regarded as *tuakana* (kin) to Māori with many *iwi/hapū* holding significant oral traditions on these species (Haami 2012; Pryor Rodgers 2017). NZ is considered a hotspot of international importance for marine mammals and seabirds, with over half of the world's species of both groups found in NZ waters (Chown et al. 1998; Gordon et al. 2010; Kaschner et al. 2011). Management of threats to megafauna is undertaken largely via the gazetting of marine mammal sanctuaries under the Marine Mammal Protection Act (1978), and fisheries restrictions under the Fisheries Act (1996); yet many locations in NZ are significantly data‐limited with respect to the information required to understand the fundamental aspects of populations' ecology and status (Schipper et al. 2008; Pott and Wiedenfeld 2017). Without this information, it is difficult to make informed decisions on the risks to populations from the range of threats that exist within NZ waters (Slooten and Dawson 1995) As a minimum, accurate information on the spatial and temporal variability in species occurrence, distribution, habitat use and population status (i.e., abundance, survival, reproductive rate) is considered critical information for species' management (Avila et al. 2018; Bestley et al. 2020). In addition, a range of human activities (tourism, shipping, coastal development, underwater noise among others) can disrupt critical behaviours (e.g., foraging, resting, nursing/provisioning young) and alter the behavioural budget of individual animals (Brakes and Dall 2016), which can have a significant impact on populations (Bejder et al. 2006; Baker et al. 2007). Thus, knowledge of the incidence and distribution of critical behaviours is also important for management.

In NZ, IK is typically considered to be encapsulated within mātauranga Māori, which can be defined as ‘Māori knowledge ‐ the body of knowledge originating from Māori ancestors, including the Māori world view and perspectives, Māori creativity and cultural practices’ (Te Ara Māori Dictionary, 2025). Mātauranga Māori is considered both a body of knowledge and a way in which knowledge is generated and may be referred to as traditional/indigenous Māori knowledge (Smith et al. 2016; Hikuroa 2017), noting mātauranga Māori contains both contemporary and traditional knowledge to reflect its ever‐evolving nature (Smith et al. 2016; Hudson et al. 2020). In this study, we use the terms Māori knowledge and mātauranga Māori to refer to knowledge generated and held by Māori that relates to the ecology of the rohe moana (*tribal waters*) of Patuharakeke a *hapū* (sub‐tribe) of the Whangārei region of NZ.

**FIGURE 1:**
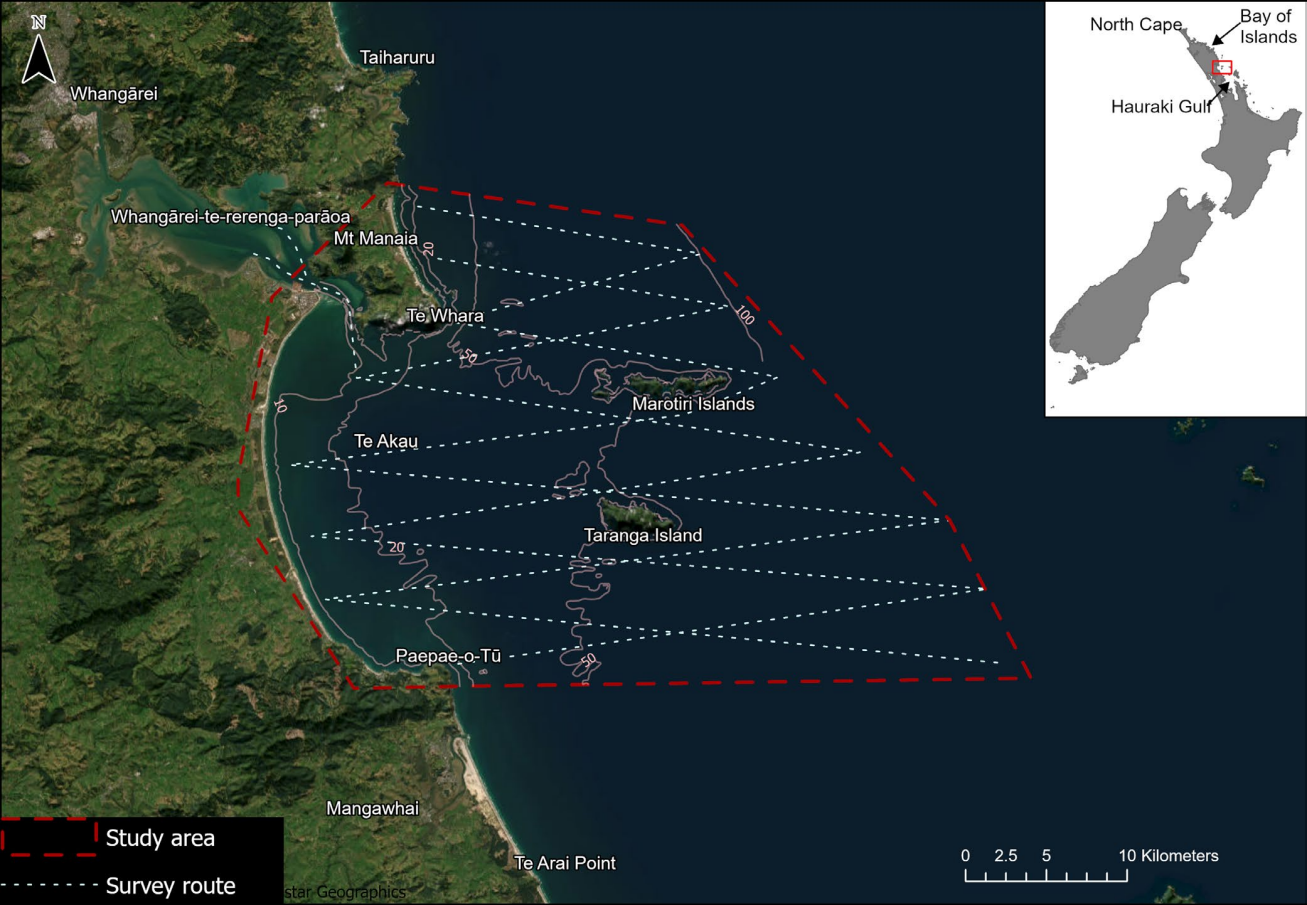
The study area in the rohe moana (tribal waters) of Patuharakeke including Te Ākau/Bream Bay and Whangārei Terenga Parāoa/Whangārei Harbour. The route surveyed during systematic vessel‐based surveys is shown along with the study area's location on the north‐east coast (between the Hauraki Gulf and North Cape) of New Zealand's North Island.

Within NZ, the north‐east of the North Island between Tikapa Moana/Hauraki Gulf and Otou/North Cape (Figure 1) is a recognised area of importance due to the high abundance of a range of threatened species (IUCN 2020). However, there have been no systematic surveys to generate the types of information required for evidence‐based management throughout the region, with prior surveys focusing on specific locations within the wider region, e.g., the Hauraki Gulf (Wiseman et al. 2011; Constantine et al. 2015; Dwyer et al. 2016), the Bay of Islands (Constantine et al. 2004; Tezanos‐Pinto et al. 2013), and off North Cape (Far Out Ocean Research, unpublished data.). Ecologically, there are no reasons to expect that these areas, should have better quality habitat for megafauna than those that have not received surveys, with similar oceanographic features, habitat types and distribution of primary productivity found throughout the region. Yet, surveyed locations are routinely held up as ‘hotspots’ for megafauna (e.g., Stephenson, Brough, et al. 2023; Stephenson, Hamilton, et al. 2023). Further, *mātauranga Māori* (Māori knowledge) in the form of place names and *pūrākau* (legends), *whakapapa* (genealogical relationships) and *kōrero tuku iho* (oral traditions) supports the idea that many discrete locations throughout the north‐east are important for these *taonga* (treasured) species.

Whangārei Terenga Parāoa/Whangārei Harbour and Te Ākau/Bream Bay are key areas that have received no formal surveys for marine megafauna. Opportunistically collected data (largely from the public) confirm the presence of a range of marine mammal species, including several threatened species such as terehu/common bottlenose dolphin ( *Tursiops truncatus* ), tohorā/Bryde's whale ( *Balaenoptera edeni brydei* ) and maki/killer whale ( *Orcinus orca* ; DOC 2023). However, these data are inadequate for providing appropriate information for the management of these species. The area is facing significant pressure from coastal development and resource use that may result in stressors upon local megafauna. These include planned extensions of the local commercial port and corresponding increases in shipping traffic and mining of sand from the sea floor, which may impact marine mammal populations (Nairn et al. 2004; Constantine et al. 2015; Forney et al. 2017). Coupled with the broad‐scale anthropogenic impacts associated with climate change (Simmonds and Isaac 2007; Peters et al. 2022) and potential impacts associated with existing local stressors (e.g., commercial fishing, habitat degradation from land‐use practices; Slooten and Dawson 1995), local megafauna may face considerable cumulative impacts, which increases the need for detailed information on population ecology.

Whangārei Terenga Parāoa/Whangārei Harbour and Te Ākau/Bream Bay are culturally, ecologically, spiritually and economically significant places to the people of Patuharakeke, a *hapū* of Whangārei. Patuharakeke have strong ties to the waters of Whangārei Terenga Parāoa/Whangārei Harbour and Te Ākau/Bream Bay with these areas considered a *taonga* handed down with reverence from *tūpuna* (ancestors; Chetham and Pitman 2014; Midwood and Chetham 2023). Patuharakeke have long‐standing associations with marine megafauna and thus, through *kōrero tuku iho*, *tikanga* (cultural protocols) and modern observations, have a wealth of knowledge on local species (Chetham and Pitman 2014; Midwood and Chetham 2023). However, such knowledge has been deliberately suppressed nationwide by historical colonial and later government authorities through mechanisms including the Tohunga Suppression Act (1907), which deliberately limited the ability of Māori communities to use and perpetuate mātauranga Māori (Ataria et al. 2018) and the Native Schools Act (1867) that prohibited the learning and use of te reo Māori (the Māori language) upon which the knowledge system depends (Broughton and McBreen 2015; Mead 2016). Additionally, Māori knowledge was systematically neglected, undervalued and under‐resourced in the face of the dominant western knowledge systems by New Zealand society during most of the 20th Century (Broughton and McBreen 2015; Ataria et al. 2018), resulting in the loss of much place‐based environmental knowledge. Thus, at local scales, it is not uncommon for mātauranga Māori to be obscured and/or missing, being unavailable to *hapū* members who may wish to draw on such knowledge to support *kaitiakitanga* (guardianship) of *rohe moana* (tribal waters).

The unavailability of knowledge from both conventional scientific surveys and indigenous knowledge has resulted in information gaps that developers may exploit to suggest that the biodiversity values of the area are low, a well‐known issue in resource management processes (Olsen et al. 2011; Cvitanovic et al. 2015). Filling these gaps are a key aspiration for the people of Patuharakeke, along with reestablishing connections with marine megafauna, revival of mātauranga Māori and capacity development for whānau interested in building technical skills to monitor populations of taonga species (Chetham and Pitman 2014).

In this study, we aim to take a holistic approach to establish a knowledge‐rich baseline on the marine megafauna of Te Ākau/Bream Bay. Specifically, we aim to (1) draw together Māori knowledge on marine megafauna within the *rohe moana* of Patuharakeke from existing sources both within and outside of the *hapū* of Patuharakeke, (2) undertake systematic surveys and combine this information with Māori knowledge to develop a comprehensive baseline of megafauna species and address the key gaps on the occurrence, spatiotemporal distribution, behaviour, and the relative importance of the area. Together, the delivery of these aims will provide Patuharakeke and their wider partners with the information, across dual knowledge systems, to care for a critical, culturally important component of their *rohe moana* and will inform ongoing resource management processes.

## 2 Materials and Methods

### 2.1 Wānanga (Shared Learning)

This study was delivered as a *hononga* (partnership) between local *hapū* (Patuharakeke) and two scientific organisations and was fully co‐developed from conception to delivery. *Wānanga* is a *te reo Māori* term that describes a process of reciprocated learning and sharing/gathering information that involves deliberation, open discussion and interpretation. In this study, we held nine *wānanga* over 2 years. An initialisation *wānanga* was held to introduce our project and socialise and gain input on our objectives with Patuharakeke members outside of our core team and the wider *whanui* (Māori community). *Wānanga* were then held alongside each of the seven vessel‐based field surveys (see below) to provide opportunities for capacity building and exchange of information across knowledge systems by weaving *wānanga* within each of our scientific surveys. A final *wānanga* shared findings back to the community. Invitations to wānanga were sent out via *panui* (notice) to all Patuharakeke members via email and the hapu's social media pages. Between fifty and 70 members of Patuharakeke and the wider *whanui* attended the project initialisation and final wānanga, while between 20 and 30 members attended the vessel‐based wānanga. Throughout the project's *wānanga*, but particularly during the project initialisation, we synthesised information that could be used to guide the design of the scientific survey. This included information on likely species presence, potential distribution and times of the year particular species may occur and be abundant.

During *wānanga*, the project brought together *mātauranga Māori* (Māori knowledge) and *kōrero tuku iho* (oral traditions) to help build a holistic picture of the importance of the study area to marine megafauna. Such information was recorded during conversations with Patuharakeke members throughout the project, rather than formal interviews. Conversations were informal and typically took place during combined *wānanga*/fieldwork or presentations on the project to the wider *whānau* (extended network/family) at Takahiwai Marae (meeting house). During vessel‐based *wānanga*, Patuharakeke members and the science team members discussed aspects of the species assemblage, ecology and habitat of the study area, often during encounters with megafauna (see below). Discussions were typically initiated by a set of broad questions relating to species occurrence, spatial and temporal distribution within the area and often resulted in the sharing of knowledge on place names, *pūrākau* (legends), associations among species and the relationship between species and the environment (i.e., depth, temperature), that can be used to inform analysis of spatial distribution (see below). In addition, guest *tohunga* (experts) from other *iwi* (tribes) were invited to attend our *wānanga* and vessel‐based field programmes. These distinguished guests took an active part in our *wānanga* and the knowledge they shared was woven into our understanding of the species and the study area. All aspects of *mātauranga Māori* gifted to the project team were recorded in detailed written notes, that were then shared back with knowledge holders to ensure accuracy.

In accordance with the *tikanga* (protocols) of Patuharakeke around sharing/disseminating *mātauranga Māori*, we obtained free, prior and informed consent from *wānanga* participants who were identified as knowledge holders. Informed consent included discussion and adherence to the principles relating to research participants and research personnel as set out in the Code of Ethics of the Association of Social Science Research of New Zealand, and confirmation that all *mātauranga Māori* would remain in custodianship of Patuharakeke to be shared according to the *hapu's* directive and according to *tikanga* and *kawa* (rules/obligations). Patuharakeke members were responsible for pooling their own *mātauranga Māori*, and thus the ethics approval processes of the scientific partner organisations was deemed unnecessary.

In addition to *wānanga*, we undertook a review of existing documentation from Patuharakeke and the wider *whānui* (e.g., Ngātiwai iwi) that has been prepared to explain the ecological significance of the area. Documents included submissions on various applications under the Resource Management Act (1991) and briefs of evidence under the Marine and Coastal Area (Takutai Moana) Act (2011). Such documentation often contains historical statements from *iwi*/*hapū* members not available to take part in our *wānanga* and provides valuable cultural information.

### 2.2 Systematic Vessel‐Based Surveys

We undertook systematic vessel‐based surveys of the wider Te Ākau/Bream Bay area using visual and acoustic methods to gather data on marine megafauna. The study area encompasses the wider Whangārei Terenga Parāoa/Whangārei Harbour and Te Ākau/Bream Bay area off the north‐eastern coast of NZ from the shore to the 100‐m depth contour (Figure 1). Seven vessel‐based line transect surveys were conducted between December 2022 and March 2024. Māori knowledge suggested there would be seasonal patterns in marine mammal occurrence. Thus, we stratified our survey effort seasonally, with at least one survey being undertaken in each season, with multiple surveys during the warmer months of the year. Each survey covered the full study area and was 3–5 days in duration. Surveys were conducted aboard a 22‐m sailing vessel with a cruising speed of 6 knots and observer height of 2.5 m.

A line transect survey design (Buckland and Turnock 1992; Strindberg and Buckland 2004) was used to determine distribution and relative density (e.g., via sighting rates) of megafauna; however, we did not use distance sampling methods to estimate true density or calculate abundance due to a low likelihood of obtaining sufficient sightings for estimating detection probabilities (Buckland et al. 2001). The study area was divided into 12 transect lines running perpendicular to the coastline in a zigzag pattern to ensure even coverage (Figure 1). Transect lines were spaced approximately 5 km apart and ran from close to shore to the 100‐m depth contour, ranging in length from 16.3 to 32.2 km. Transect spacing was designed to maximise coverage of the study area and all key habitat types, while allowing for a single survey to be completed within a single ‘good weather’ window (typically 3–5 days). Surveys were conducted during daylight hours and in sea conditions of Beaufort wind scale < 4, swell of ≤ 1 m and good visibility. All effort (survey track) data were continuously saved in the CyberTracker app (CyberTracker Software Ltd.) on an Android tablet.

Observations of all megafauna except seabirds were made via a continuous scanning method (Mann 1999), using both the naked eye and binoculars containing rangefinder reticules and a compass. Two observers were placed on the vessel's bow, with each scanning the area from the bow to 90 degrees to the port or starboard side of the vessel, respectively, in a single‐observer protocol (i.e., single observer per bow/starboard sector; Buckland et al. 2001). Observers were rotated in 40‐min intervals. Observers scanned the area for megafauna cues, including blows, splashes, fins or seabird activity. Upon detection, the species name, group size, behaviour, compass bearing and distance from the observation platform were recorded. Compass bearings and reticules were used to accurately position the location of the sighting to enable better matching with environmental data for spatial modelling (see below).

For high‐priority species (terehu/bottlenose dolphins, tohorā/Bryde's whales, mautai/false killer whales, maki/killer whale), survey effort was paused, and the species was approached for photo‐identification (photo‐ID) of individuals. All megafauna sightings data were entered into a purpose‐built programme within the CyberTracker app. Kororā/little penguins ( *Eudyptula minor* ) were sampled using this method due to an interest in obtaining all sightings of this species due to their cultural importance. For the other seabirds, two seabird observers were placed on the bow of the vessel to undertake seabird counts. A strip‐transect method (Tasker et al. 1984) was applied, with an effective strip width of 200 m (100 m each side of the vessel) to reliably identify all seabird species. Strip width was estimated using Heinemann's (1981) method. Seabird observers counted all species that occurred within the strip width during 10‐min intervals, followed by a 10‐min break and were also rotated in 40‐min intervals. In most cases, seabirds were able to be assigned to species level; however there are considerable similarities between some species that may co‐occur in the study area and in these cases, seabirds were identified to shared species group (e.g., prion spp.). Data on the start, end and seabirds observed during each seabird count were entered directly into the CyberTracker app along with observer ID and survey conditions (sea state, swell etc.).

To further document species present within the study area we made 10‐min acoustic recordings, using a custom‐made hydrophone array deployed from the stationary (with engine shut down) research vessel. The array was suspended between 10 and 20 m below the hull of the vessel to minimise masking from the vessel's presence. An Edirol R4 digital acoustic recorder sampling at 48 kHz was used to make the 10‐min acoustic recordings, which were saved on an internal memory card. Recordings were made at approximately five nautical mile intervals along the transect lines and when sea conditions were at Beaufort wind scale < 4 and no rain was present. The recording was monitored in situ using headphones and any vocalising marine mammals noted, along with any obvious anthropogenic/natural noise (e.g., vessel noise/swell breaking ashore). The details of each recording including time, date, geographic coordinates and any of the above‐mentioned notes were saved in the Cybertracker app.

In addition to the data collected using systematic surveys, opportunistic marine mammal sightings were obtained from other research projects operating in the area to provide further insights into marine mammals in the study area. These sighting records encompassed the same sighting information and photo‐ID methods as described above, but did not occur during the windows prescribed for the surveys under this project (though they largely occurred over the same time period). Opportunistic data were collected using the same criteria as the systematic surveys with the exception that survey effort was more directly targeted to areas where key species were likely to be found, rather than following a line transect design. These data were integrated with systematically collected data from several of the analyses as discussed below.

### 2.3 Photo‐Identification

We used photo‐identification (photo‐ID) approaches to assess site fidelity, residency patterns and to collect data on population demographics for common species of marine mammals, where identifying features would allow the identification of individual animals. These species included tohorā/Bryde's whale, terehu/bottlenose dolphin and mautai/false killer whale ( *Pseudorca crassidens* ). Standard photo‐ID methods (e.g., Würsig and Jefferson 1990) were applied. Briefly, the research vessel was manoeuvred so as to be travelling parallel to the group's course and was gently positioned within a distance that enabled good quality dorsal fin photographs to be obtained. Primary identification features included notches on or adjacent to the leading or trailing edge of the dorsal fin. Dorsal fin images were graded according to the likelihood of successful resightings and matched following procedures established by Zaeschmar et al. (2020). Only individuals classed as ‘very distinctive’ or ‘distinctive’ and images of excellent or good quality were included in the analysis.

For each encounter, the number of unique individuals was calculated and matched against existing catalogues of individuals to determine the number of resighted individuals (i.e., previously photographed during this study) detected. The total number of individuals with uniquely identifying dorsal fin marks enables an estimate of the minimum number of the species that uses the study area and the resighting rate provides an estimate of site fidelity (i.e., how often individuals are present). As this study undertook the first photo‐ID sampling of coastal bottlenose dolphins in the study area, a new catalogue was created with ‘new’ individuals iteratively added as they were encountered. Photo‐ID images for mauitai/false killer whales were matched against the New Zealand false killer whale photo‐identification catalogue (Zaeschmar et al. 2014). Images of Bryde's whale were matched against the University of Auckland's NZ Bryde's whale catalogue. All photo‐ID data will be integrated into wider studies on the population biology of these species using mark‐recapture analysis.

### 2.4 Species Occurrence

Rates of occurrence (i.e., sightings per unit of effort, SPUE) of all megafauna except seabirds were calculated using information on the number of sightings obtained during each survey, standardised by the recorded kilometres of survey effort. Sightings per kilometre of effort were calculated for each species encountered and for each of the seven surveys. A ‘survey’ represents a full, systematic sample of the entire study area and thus a SPUE estimate was calculated for each monthly survey, rather than for discrete days during a survey. Sightings and effort were then aggregated to the austral season when multiple surveys occurred in a given season (e.g., December, March) and combined over the full study period to provide an estimate for a general rate of occurrence for each species over the study area across all seasons. Such indices of relative abundance provide useful insights into the spatial and temporal occurrence patterns of mobile marine species (Weir et al. 2007; Dwyer et al. 2016; Melly et al. 2018; Dolman et al. 2014; Lennert‐Cody et al. 2018; Stephenson, Hamilton, et al. 2023; Putra et al. 2025), that allow comparison across temporal scales or to other areas with similar survey methodology under a range of assumptions (Lennert‐Cody et al. 2018).

### 2.5 Species Distribution Modelling

Analyses of distribution and habitat use for the most commonly occurring (more than 20 occurrences) megafauna species were undertaken using a species distribution modelling (SDMs) approach (Guisan and Thuiller 2005; Elith and Leathwick 2009). For marine mammals, we fit SDMs using occurrence and background data (sometimes referred to as absence/pseudo‐absence data; Phillips et al. 2009) obtained during both systematic surveys and from opportunistic encounters. A database of background data was created using recorded survey tracks from our systematic surveys. For each survey day, daily background data were randomly sampled in a 2000 m buffer around our daily survey track—the average distance at which we can make reliable detections across all species encountered in this study. Background points with unique date stamps were pooled into a database for the full study period, and environmental data were extracted for each point. For seabirds, individual seabird counts were used to generate SDMs with occurrences being characterised by the presence of a particular species during a 10‐min count. Counts where the given seabird species was not recorded were used as absences and are thus representative of true absences.

Species occurrence and background/absence data were matched with spatially and temporally co‐located environmental data using the *extract* function of the *terra* package (Hijmans 2023) in R 4.3.2 (R Core Team 2023). Temporally dynamic variables were extracted at a monthly scale using the dates attributed to each occurrence/background data point. Environmental datasets consisted of static spatial layers representing sea floor characteristics (e.g., depth, slope, terrain characteristics) and average tidal current speed. Dynamic (i.e., temporally variable) environmental data included sea surface temperature (SST), primary productivity (chlorophyll *a* concentration, CHLA), and measures of turbidity—particulate backscatter (BBP) and light irradiance at the seafloor (EBED). Dynamic variables were sourced at monthly resolution (the highest temporal resolution with continuous coverage for each survey) from the Seas Coasts Estuaries New Zealand (NIWA‐SCENZ) data repository (Pinkerton et al. 2022) hosted by NIWA. NIWA‐SCENZ provides a broad range of satellite remote sensing data that have been processed to provide the best available information on coastal water conditions/quality for the NZ coast (Pinkerton et al. 2022). However, some datasets (e.g., CHLA) provide only proxy information for biological processes and thus caution should be taken when interpreting relationships between these variables and the distribution of wide‐ranging species. A dynamic variable for horizontal gradient in SST was also calculated based on mean monthly SST variables. All environmental datasets were represented as gridded raster layers with 500 m × 500 m resolution. See Table A1: Appendix S1 for a full list and description of the available environmental data. A categorical variable ‘season’ was also included to represent occurrences/background data for the warm season (December—March) and cool season (June—September) and was used to account for any unexplained seasonal variation in the models. ‘Season’ was used instead of ‘month’ due to low data coverage within individual monthly surveys and subsequent predictions being generated into poorly sampled environmental space (Elith and Leathwick 2009).

We used random forests (RF) (Breiman 2001), to model and predict species probability of occurrence for the study area. Random forests were fitted using the tuneRF function of the package RandomForest (Liaw and Wiener 2002) in R, with a binomial response variable and with 1500 trees. The performance and inference from RF models have limited susceptibility to correlation among predictor variables (Breiman 2001). However, highly correlated variables (correlation coefficient > 0.85) were removed to prevent overfitting (Elith et al. 2006). A list of non‐correlated environmental variables and the ‘season’ categorical variable was supplied as predictor variables for the SDMs. For each species, RFs were trained using a randomly selected training dataset consisting of two‐thirds of the occurrence data and a randomly selected equal number of background data points. The remaining third of occurrences and an equal number of randomly selected background data were retained as withheld evaluation data. This process was repeated 100 times for each species using a bootstrapping approach, with a RF model being fit for each iteration and subsequent generation of spatial predictions and uncertainty (standard deviation of 100 spatial predictions).

Model validation was undertaken by comparing predictions from models tuned using the training dataset with observations from the withheld evaluation data set using the Area Under the Receiver Operating Curve (AUC) and the True Skills Statistic (TSS) metrics (Allouche et al. 2006). The model tuning and evaluation process was repeated 100 times, and the mean and standard deviation of the AUC and TSS values were used to determine the performance of each model.

Spatial predictions of species' probability of occurrence were generated for an approximately 80 × 50 km area centred on our study area using gridded environmental data at 500 m cell resolution with the final predictions being an average of the 100 model runs for each species. An area slightly larger than the model tuning area (i.e., survey area) was used to determine any hotspots of species distribution immediately outside of Patuharakeke's key area of interest which may guide future work. To capture any seasonal patterns in occurrence, predictions were made for two broad seasons (warm/cool season). April, May, October and November were excluded from the predictions as no surveys were undertaken during these months. The temporary dynamic variables (SST, BBP, CHL, EBED) were averaged across months within each broad season to generate seasonally averaged prediction data frames that were merged with the additional static environmental data sets (Table A1: Appendix S1).

An evaluation of the importance of the environmental predictor variables to each species' SDM can provide insights into the habitat preferences of each species (Brough et al. 2023). Thus, the relative importances of each environmental predictor were calculated from the RF models using a standardised calculation of the variable importance measure (VIM). The importance of each environmental variable predictor *p* in a RF model, $R_{p}^{2}$, is given by (Ellis et al. 2012):

$$R_{p}^{2}=\frac{R^{2}I_{p}}{\sum_{p^{\prime }}I_{p^{\prime }}}$$

where $I_{p}$ is the accuracy importance of each predictor in a forest, and *R* ^2^ is the proportion of variance explained by the forest. The goodness of fit, *R* ^2^, is partitioned among the predictors in proportion to their accuracy importance, $I_{p}$. The accuracy importance ($I_{p}$) is standardised by the densities across the raw importance from each split in each tree (for each variable *p*) and normalised such that they sum to $R_{p}^{2}$ (Ellis et al. 2012).

### 2.6 Acoustic Analyses

PAMGuard (v2.02.09 CORE) (Macaulay and Gillespie 2022) was used to automatically detect cetacean calls. The acoustic recordings were clipped to a 10‐min duration using the package tuneR (Ligges and Krey 2024) in R and PAMGuard's Whistle and Moan detector (Gillespie et al. 2013) was configured to detect odontocete whistle contours based on settings used in similar environments and with similar species assemblages (Rankin et al. 2017; Jones et al. 2021). PAMGuard's ROCCA classifier was then used to identify potential species (Oswald et al. 2007) using the ‘Temperate Pacific’ classifier (Oswald and Yack 2015). with any detections classified as “Ambiguous” excluded (69%) along with contours with a duration less than 0.2 s (27%).

To verify the accuracy of the detection process, 12% of the full dataset (Insley et al. 2021; Pérez Tadeo et al. 2023), was randomly selected for manual screening and subsequent calculation of false positive/negative rates for the detection process (see Appendix 3: Appendix S1 for more detail).

## 3 Results

### 3.1 *Mātauranga* Māori From wānanga

**FIGURE 2:**
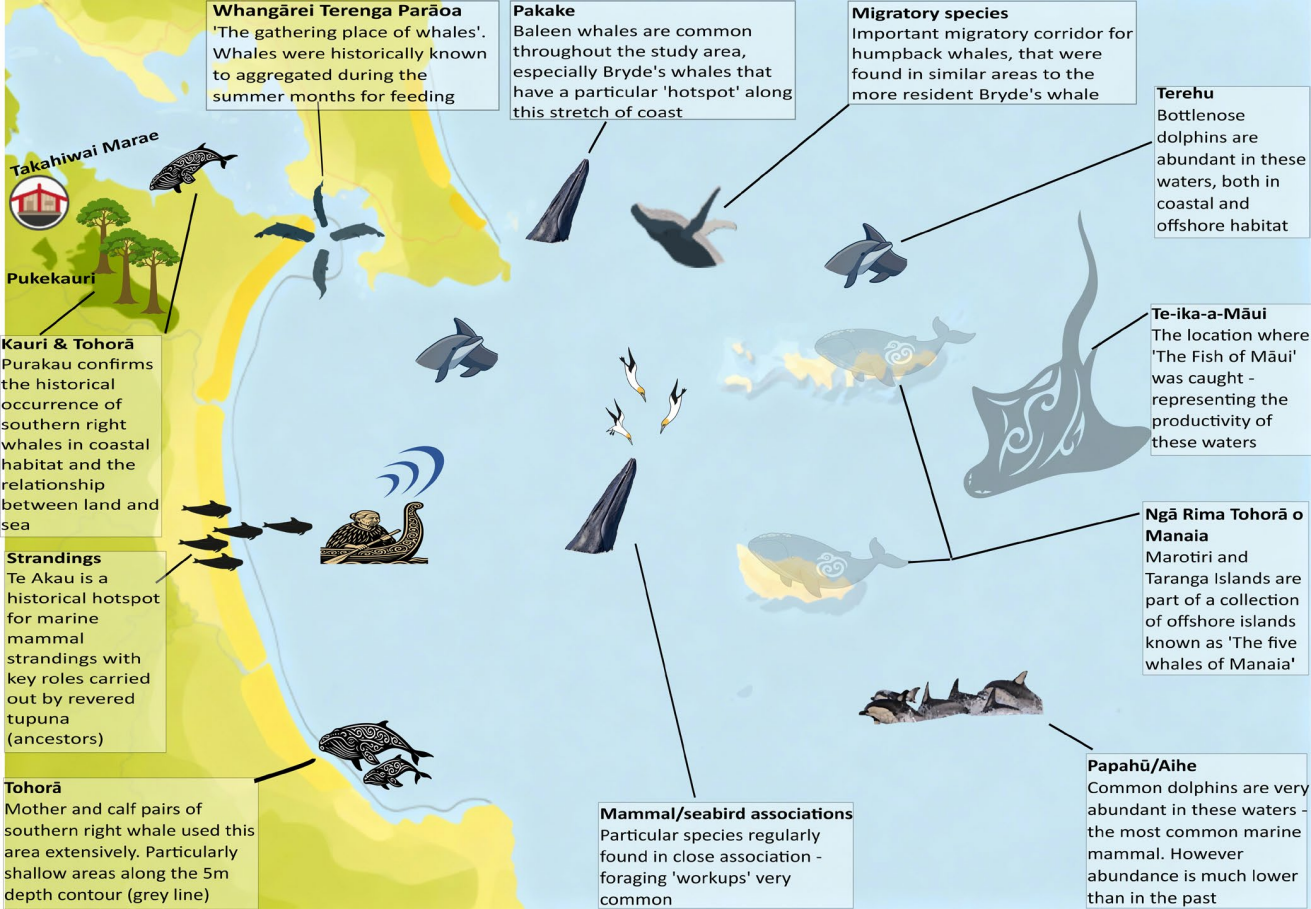
Schematic diagram depicting the key findings from wānanga where mātauranga Māori (Māori knowledge) on the marine megafauna of the study area was gifted to the study.

Māori knowledge from a range of sources was gifted to our *wānanga* (Figure 2). These included various components of *mātauranga Māori* (Māori knowledge) such as *kōrero tuku iho* (oral traditions) and *pūrākau* (legends) concerning (largely) marine mammals and the relationship between the people of Patuharakeke and these species, and the ecology of the study area.

### 3.2 Place Names

There are various *mātauranga* relating to the meaning of the harbour's name that are shared and valued among local *iwi* (*tribes*), including Patuharakeke. One interpretation of Whangārei Terenga Parāoa follows the direct translation to “the gathering place of whales”. It is said that Parāoa (sperm whales, *Physeter macrocephalus* ) would visit the waters of the study area to feed during summer when the waters are warm and productive. However, contemporary (i.e., modern/current) Māori knowledge shared during *wānanga* suggests the study area is not a typical habitat for sperm whales (i.e., shallow, coastal habitat) and thus Parāoa may refer to other species of whale, or alternative meanings for the harbour's name (e.g., a gathering place of chiefs) may be more plausible. In any case, the potential for seasonal patterns in whale occurrence was woven into our survey programme with a higher frequency of surveys during the warmer season and by the use of environmental variables (SST, SSTGradient, CHLA) that may be correlated with the seasonal occurrence of whale species with the SDMs (Table A2: Appendix S1).

Within and immediately to the north and east of the study area lie many islands that include Aotea (Great Barrier Island), Hauturu (Little Barrier Island), Tūturu (Sail Rock), Marotiri (often referred to as “The Chicks”) and Taranga (often referred to as “The Hen”) and the Motu Kino and Pokohinu (Mokohinau) Islands and Aorangi and Tawhiti Rahi (the Poor Knights Islands). These island groups make up what are known in oral tradition as ‘Ngā Rima Tohorā o Manaia*’* (the five whale families or groupings of Manaia). While the specifics of the oral traditions around this name were not available, the naming of the dominant geographic features in and around Patuharakeke's tribal waters exemplifies the deep‐rooted connection between marine mammals and Patuharakeke.

All of the islands in the Marotiri cluster are named after the legendary Polynesian figure Māui and his *whānau* (extended family), with Taranga being the mother of Māui. Ngātiwai (iwi) tradition integrates these names with the famous *pūrākau* of Māui catching *Te‐ika‐a‐Māui* (the North Island of NZ), with the action of ‘catching the fish’ occurring within the waters of this study area. It is said that the highly productive waters of the area that sustain such abundance were the key to catching *Te‐ika‐a‐Māui* and are linked with the understanding of the importance of the area for numerous *taonga* (treasures) (including megafauna). When Māui landed *te ika*, it was attacked and cut into pieces by his brothers. The pieces that fell from *te ika* were called “Ngā Unahi me ngā Taratara o Te Ika roa o Maui” (the scales from the fish of Māui) and became the many islands and rocky outcrops throughout the tribal waters of Ngātiwai and Patuharakeke, acting as a reminder of the productivity of these waters.

### 3.3 Tohorā and Kauri

A *pūrākau* that is significant to Patuharakeke is the connection between the Kauri tree ( *Agathis australis* ) and Tohorā (H. Parata, pers. comm). It is said that long before humans, Kauri and Tohorā (southern right whale in this context, *Eubalaena australis* ) were brothers who lived together on the *whenua* (land). Tohorā loved to visit the *moana* (ocean) and one day, Tohorā had a calling to live in the *moana*. Excited, Tohorā asked Kauri to join him, but Kauri could not leave the *whenua*. Instead, Kauri gave Tohorā his blessing to follow his calling. Tohorā gifted Kauri the scales of his skin to allow him to be protected and grow tall. In return, Kauri gifted Tohorā his oil to provide him with extra warmth on his travels through the ocean. Kauri was sad to see Tohorā leave, so he made his way to the top of the ridges and grew to the top of the canopy to see Tohorā voyaging through Aotearoa. Tohorā would swim close to the coast and breach so that his brother could see him follow his calling. This *pūrākau* is commonly told by northern tribes and is reflected locally through Patuharakeke *pūrākau* identifying the strong connections between the Pukekauri range behind the local *Marae* (meeting house) and Whangārei Terenga Parāoa. Patuharakeke sees the main *awa* (river), Takahiwai, as the connection between Pukekauri and Whangārei Terenga Parāoa. *Kōrero* at *wānanga* suggested the local adaptation of this legend affirms tohorā/southern right whales would have been once common in the tribal waters of Patuharakeke, and that their distribution would have been close to shore, in sight of the local Pukekauri range.

### 3.4 Species Specific Insights

During *wānanga*, Patuharakeke members and guest experts in Māori knowledge (R. Stewart, pers. comm), provided a range of insights specific to individual species, including important information for both present‐day and historical megafauna populations.

Papahū/Aihe, short‐beaked common dolphins ( *Delphinus delphis* ) are abundant in the waters of Te Ākau, Bream Bay. However, the present‐day abundance of the species is believed to be much lower than it was historically. Similarly, terehu, bottlenose dolphins ( *Tursiops truncatus* ) had historically high occurrence in both the offshore and coastal areas within the rohe moana of Patuharakeke.

Similarly, the area is an important habitat for pakake (also referred to as tohorā by some *whānau*), the rorquals (genus Balaenoptera), and especially Bryde's whale ( *Balaenoptera edeni* ) which show a preference for some near‐shore habitats that overlap with known key areas for more migratory baleen whale species. An area of particular importance for whale species can be found between Te Whara, Bream Head and Taiharuru (Figure 2).

Tohorā, the New Zealand Southern Right Whale ( *Eubalaena australis* ) was once common in the rohe moana of Patuharakeke. They would begin to move north in the late spring to a historical foraging ground that lay east southeast (ESE) of Rangitāhua, the Kermadec Islands, on the Lau‐Colville seamount chain. Tohorā cow/calf pairs were strongly associated with shallow areas inside the 5 m depth contour off sandy beaches. Additionally, the study area was also an important migratory corridor for pakake/Paikea, the Oceania humpback whales ( *Megaptera novaeangliae* ). These whales passed through the area in significant numbers during their southward migration between their tropical breeding grounds and the Antarctic, being particularly common in late spring. (R. Stewart, pers. comm).

Whales and seabirds are closely associated with one another, with certain seabirds and their preferred choice of prey being strongly linked with the occurrence of different whale feeding strategies. (R. Stewart, pers. comm).

### 3.5 Existing Documentation

Throughout *wānanga*, we were given multiple examples of the close and esteemed relationship between members of Patuharakeke and marine mammals that had been recorded in existing documentation. The following quotes are excerpts from Midwood and Chetham (2023) that exemplify this relationship.

“Whales are a key taonga (treasured) species, typically associated with our rohe moana (tribal waters) and takiwā (district/territory). They are viewed as a “tuakana” (kin) to us. In former days the waters off our shores abounded with species of both seal and whale, and in recent times, over the last hundred or so years much interest has been created by the occasional visits and even strandings of these creatures. For our people, particular thought is given to the possible portent of what these visits and strandings may indicate because these creatures are regarded as the lineal descendants of the tribal taniwha of the ancient past.” (Midwood and Chetham 2023, 5).

“In Patuharakeke lore, it is told that when a whale stranded in our waterways a practice of old would be for the kuia of the tribe to embark on a waka, and karanga or call to the whale and guide its safe passage out to sea again. Wāhi tūpuna associated with this are Te Waiparāoa at Mangawhati and Te Hōpua/Ngātītī. This role would also have been performed by tohunga (experts) of the tribe and at times when maleficent taniwha would endeavour to overcome people and the tohunga and his incantations would be at work either placating the taniwha or capturing or weakening the creature.” (Midwood and Chetham 2023, 12).

Historically, the study area has been a stranding hotspot for marine mammals and Patuharakeke and Ngātiwai *whanui* (Māori community) are the first responders for these incidents. In the event of a stranding, Patuharakeke is called and actively seeks to refloat stranded marine mammals. If all refloating attempts are unsuccessful, the *hauhake* (traditional harvesting) process is actioned in the presence of esteemed *kaumatua* (elders) and *tōhunga*. The *tikanga* (cultural protocols) and cultural practice of the tohorā *hauhake* process had been lost over time as an effect of colonisation, but due to a mass stranding event of ūpokohue/pilot whales ( *Globicephala melas* ) in Te Ākau in 2006, tohorā *hauhake* has become revitalised and is now a common practice for stranded/deceased marine mammals in Patuharakeke tribal waters. The naming *tikanga* is a significant part of the process, where stranded whales are named to enhance the *mana* (respect/strength) of the *tupuna taonga* (treasured ancestor) and to carry on the *whakapapa* (genealogical relationships) of the *taonga* post‐stranding. The revitalisation of *hauhake* tohorā has inspired new generations to regain lost *mātauranga* that once highlighted the interconnected relationship of tohorā and Patuharakeke people (Chetham and Pitman 2014; Midwood and Chetham 2023).

#### 3.5.1 Vessel‐Based Surveys

There were 7 surveys between December 2022 and March 2024, resulting in 27 discrete survey days, with a total of 1537.5 km of line transects covered (Table A3: Appendix S1), with effort distributed throughout the study area (Figure A1: Appendix S1) Most systematic survey effort occurred during the summer (13 days and 686.9 km of effort), with the lowest effort in winter (3 days and 195.5 km of effort; Table A2: Appendix S1). With respect to the broad (warm/cool) seasons used for predicting distribution, effort was also skewed towards the warmer season, although a good representation of cool season conditions was sampled (combined 427.4 km of effort). However, some surveys that were used to calculate seasonal (i.e., winter, spring) sighting rates had low effort compared to summer (see discussion). A total of 223 10‐min seabird counts were undertaken during the seven surveys and were widely distributed throughout the study area.

Eight species of marine mammals were encountered throughout our surveys. Each species was recorded both during systematic surveys and opportunistic encounters except for maki/killer whale and blue whale ( *Balaenoptera musculus* ) that were seen during systematic surveys only and upokohue/long‐finned pilot whale that were seen during opportunistic encounters. Aihe/common dolphin were the most commonly occurring species (41 sightings) and were encountered over all seasons with group sizes varying between 5 and ~250 individuals (median = 25). Other commonly occurring species included tohorā/Bryde's whale (27 sightings, including 43 individuals, median group size = 1), coastal and oceanic terehu/bottlenose dolphins (33 and 40 sightings respectively) and mautai/false killer whales (36 sightings). Oceanic bottlenose dolphins are considered a different ‘ecotype’ and management unit in NZ waters and can be differentiated from coastal bottlenose by their larger size, darker colour and presence of cookie cutter shark scars (Zaeschmar et al. 2020). Group sizes for coastal bottlenose dolphins ranged from 2 to approximately 100 (median = 22.5), and ranged between 20 and ~250 (media*n* = 150) and 50 to ~150 (median = 80) for oceanic bottlenose and false killer whales respectively.

Four species of elasmobranch (sharks and rays) were seen during systematic surveys (Table A3: Appendix S1) with oceanic manta rays (*Mobula birostris*; 15 sightings) and mangōpare/hammerhead shark ( *Sphyrna zygaena* ; 11 sightings) sightings being the most frequently encountered species. Both species occurred only during summer. Kororā/Little penguin was the most commonly encountered of all megafauna sampled using continuous scanning with 104 sightings (Table A3: Appendix S1).

Twenty‐four seabird species or species complexes were observed in the study area across all surveys (Table A4: Appendix S1). Pakahā/fluttering shearwater ( *Puffinus gavia* ) was the most commonly occurring species, being observed in 36.8% of the total seabird counts and across all seasons. Other commonly occurring species included rako/Buller's shearwater (*Ardenna bulleri*), tākapu/Australasian gannet ( *Morus serrator* ), kuaka/northern diving petrel ( *Pelecanoides urinatrix* ), toanui/flesh‐footed shearwater (*Ardenna carneipes*), tītī/Cook's petrel (grouped with Pycroft petrel) (*Pterodroma* spp.) and takahikare/white‐faced storm petrel ( *Pelagodroma marina maoriana* ). Ōi/grey‐faced petrel (*Pterodroma gouldi*), cape petrel ( *Daption capense* ), Toroa/black‐browed albatross ( *Thalassarche melanophris* ), and skuas (*Stercorarius* spp.) were rarely encountered, occurring in 0.4% (former three species) and 0.9% (both skua taxa) of total counts respectively.

#### 3.5.2 Photo‐Identification

Photo‐ID was carried out during 84% of encounters with coastal terehu/bottlenose dolphins (*n* = 21). A total of 149 distinct individuals were identified. Of these, 73.1% (*n* = 109) were recorded on more than one occasion (range 1–7, median = 2) and 39.6%, (*n* = 59) were identified over more than one year (range 1–3). Calves and neonates were recorded during 71.4% of encounters (*n* = 15) and were sighted in all months. There were 41 sightings of 27 individual calves and a single sighting of a neonate. Repeat close associations of the same adult with a calf were observed in 28 cases (range 1–4, median = 1).

For mautai/false killer whales, photo‐ID was carried out during all encounters and a total of 134 individuals were photo‐identified. Photo‐ID results from this study have been integrated within a wider project on the demographic parameters for New Zealand false killer whales. Preliminary population abundance for this population has been estimated as 127 (95% CI = 114–141) using mark recapture (Zaeschmar et al. 2022). Photo‐ID was carried out during all 23 encounters with tohorā/Bryde's whales, resulting in the identification of 14 individuals of sufficient distinctiveness and image quality to be added to the NZ Bryde's Whale Photo‐identification Catalogue. Of these 14 individuals, 4 were resighted at least once with one individual sighted on three occasions, and another 2 individuals sighted in different years (range = 1–634 days, median = 4.5).

#### 3.5.3 Species Occurrence

Using standardised data from systematic surveys, most megafauna species exhibited seasonal variability in occurrence (Table 1). Monthly sighting rates for Aihe/common dolphin ranged from a low of 0.009 km^−1^ in September to 0.027 km^−1^ in January (mean = 0.020 km^−1^). Terehu/coastal bottlenose dolphins were sighted during all survey months except September and had monthly sighting rates ranging from 0 km^−1^ in June and September to 0.031 km^−1^ in January (mean = 0.011 km^−1^). Monthly sighting rates for tohorā/Bryde's whales ranged from 0 km^−1^ in June to 0.019 km^−1^ in March (mean = 0.013 km^−1^). Sightings rates for terehu/oceanic bottlenose dolphins and mautai/false killer whales suggest the species are present in the warm season only. For kororā/little penguin, monthly sighting rates ranged from 0.017 km^−1^ in September to 0.305 km^−1^ in March (mean = 0.136 km^−1^).

**TABLE 1:** Monthly sighting rates of the most frequently occurring marine mammals generated from systematic line transect surveys.

	Effort (km)	Sightings per km (Number of sightings)				
		Terehu/Coastal bottlenose dolphin	Terehu/Oceanic bottlenose dolphin	Aihe/Common dolphin	Mautai/False killer whale	Tohorā/Bryde's whale
December	461.3	0.004 (*n* = 2)	0.004 (*n* = 2)	0.017 (*n* = 8)	0.002 (*n* = 1)	0.011 (*n* = 5)
January	225.6	0.031 (*n* = 7)	0	0.027 (*n* = 6)	0	0.018 (*n* = 4)
March	423.1	0.019 (*n* = 8)	0.014 (*n* = 6)	0.024 (*n* = 10)	0.007 (*n* = 3)	0.019 (*n* = 8)
June	195.5	0	0	0.026 (*n* = 5)	0	0
September	231.9	0	0	0.009 (*n* = 2)	0	0.013 (*n* = 3)
Total	1537.4	0.011 (*n* = 17, SE = 0.005)	0.005 (*n* = 8, SE = 0.002)	0.020 (*n* = 31, SE = 0.003)	0.003 (*n* = 4, SE = 0.001)	0.013 (*n* = 20, SE = 0.003)

There was marked seasonality in occurrence for some seabird species (Table A4: Appendix S1). For example, takoketai/black petrel ( *Procellaria parkinsoni* ) and rako/Buller's shearwater did not occur during winter surveys (June), while the three albatross species were present in September and December only. Several species occurred only during surveys in the cooler months of the year (Arctic skua, grey‐faced petrel, cape petrel). In contrast, the occurrence of tākapu/Australasian gannet and pakahā/fluttering shearwater was broadly similar across the seasons and fluttering shearwaters were common across all months (Table A4: Appendix S1).

#### 3.5.4 Distribution

**FIGURE 3:**
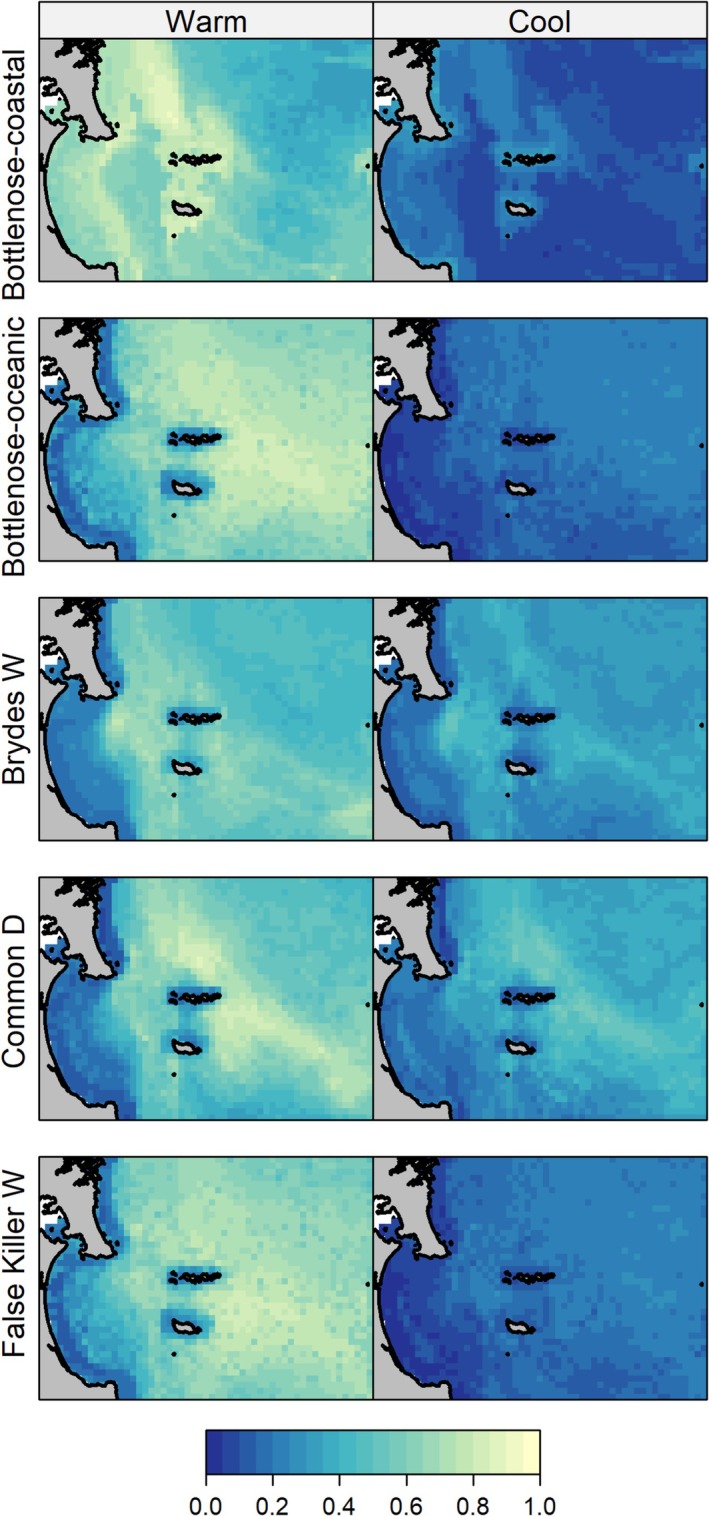
Seasonal predictions of the probability of occurrence from species distribution models for five marine mammals: coastal bottlenose dolphins (Bottlenose‐coastal), oceanic bottlenose dolphins (Bottlenose‐oceanic), Bryde's whale (Brydes W), common dolphins (Common D), and false killer whales (False Killer W), for warm (December–March) and cool (June–September) conditions.

SDMs performed well for each of the five most commonly occurring marine mammal species (AUC > 0.7, Table 2). There were strong seasonal differences in the probability of occurrence for all marine mammals, with a higher probability for all species during the warmer months of the year (Figure 3). For coastal terehu/bottlenose dolphins, predictions for the warm season revealed a high probability of occurrence throughout the northern and nearshore components of the study area including Whangārei Harbour and Te Ākau/Bream Bay. The probability of occurrence was considerably lower during the cooler months of the year. For tohorā/Bryde's whales, a high probability of occurrence during the warm season was predicted throughout the central and eastern components of the study area, with particular hotspots between Te Whara/Whangārei Heads and the Marotiri Islands, in the south‐eastern part of the study area towards Hauturu, and within Te Ākau/Bream Bay itself (Figure 3). Although tohorā/Bryde's whales are predicted to be less common during the cool season, the cool‐season predictions reveal some moderate probability of occurrence in the Te Ākau/Bream Bay area, south of Te Whara/Whangārei Heads. Predictions for common dolphins revealed distinct, seasonal differences in the probability of occurrence within the study area (Figure 3). During the warm season, a high probability of occurrence was predicted for the northern and eastern components of the study area. Hotspots were notable north and east of the Marotiri and Taranga Islands (Figure 3). In the cool season, an area of moderate to high probability of occurrence was predicted for deeper waters beyond the island groups (Figure 3).

**TABLE 2:** Model evaluation for species distribution models for the five most commonly occurring marine mammals and nine most common seabirds.

Species	Group	AUC (SD)	TSS (SD)
Terehu/Coastal bottlenose dolphin	Marine mammal	0.89 (0.05)	0.73 (0.10)
Terehu/Oceanic bottlenose dolphin	Marine mammal	0.89 (0.04)	0.72 (0.09)
Tohorā/Bryde's whale	Marine mammal	0.75 (0.04)	0.51 (0.16)
Aihe/Common dolphin	Marine mammal	0.74 (0.07)	0.47 (0.11)
Mautai/False killer whale	Marine mammal	0.89 (0.05)	0.71 (0.10)
Tākapu/Australasian gannet	Seabird	0.55 (0.05)	0.20 (0.07)
Rako/Buller's shearwater	Seabird	0.68 (0.05)	0.36 (0.08)
Tītī/Cook's & Pycroft's petrel	Seabird	0.83 (0.04)	0.60 (0.07)
Kuaka/Diving petrel	Seabird	0.85 (0.08)	0.67 (0.12)
Tītī wainui/Prion spp.	Seabird	0.79 (0.09)	0.58 (0.13)
Toanui/Flesh‐footed shearwater	Seabird	0.72 (0.04)	0.43 (0.07)
Pakahā/Fluttering shearwater	Seabird	0.68 (0.05)	0.38 (0.07)
Takahikare/White‐faced storm petrel	Seabird	0.66 (0.08)	0.35 (0.11)
Kororā/Little penguin	Seabird	0.89 (0.03)	0.65 (0.06)

*Note:* Models are evaluated by area under the receiver operating curve (AUC) and true skill statistics (TSS). Values represent mean scores (and associated standard deviation, SD) across 100 model runs evaluated with withheld data.

Predicted distribution for oceanic terehu/bottlenose dolphins and mauitai/false killer whales is suggestive of the strong seasonal presence of these species in the study area, with very low probability of occurrence during the cool season (Figure 3). Additionally, predictions for the two species are highly similar which is unsurprising given they are commonly found together. During the warm season, high probability of occurrence for both species was predicted throughout the northern and eastern components of the study area, between the Mokohinau and Taranga/Marotiri Island groups, with hotspots offshore and to the north of the Marotiri Islands. Areas with low probability of occurrence included the inshore western region of the study area, and the waters close to the Marotiri and Taranga Islands (Figure 3).

**FIGURE 4:**
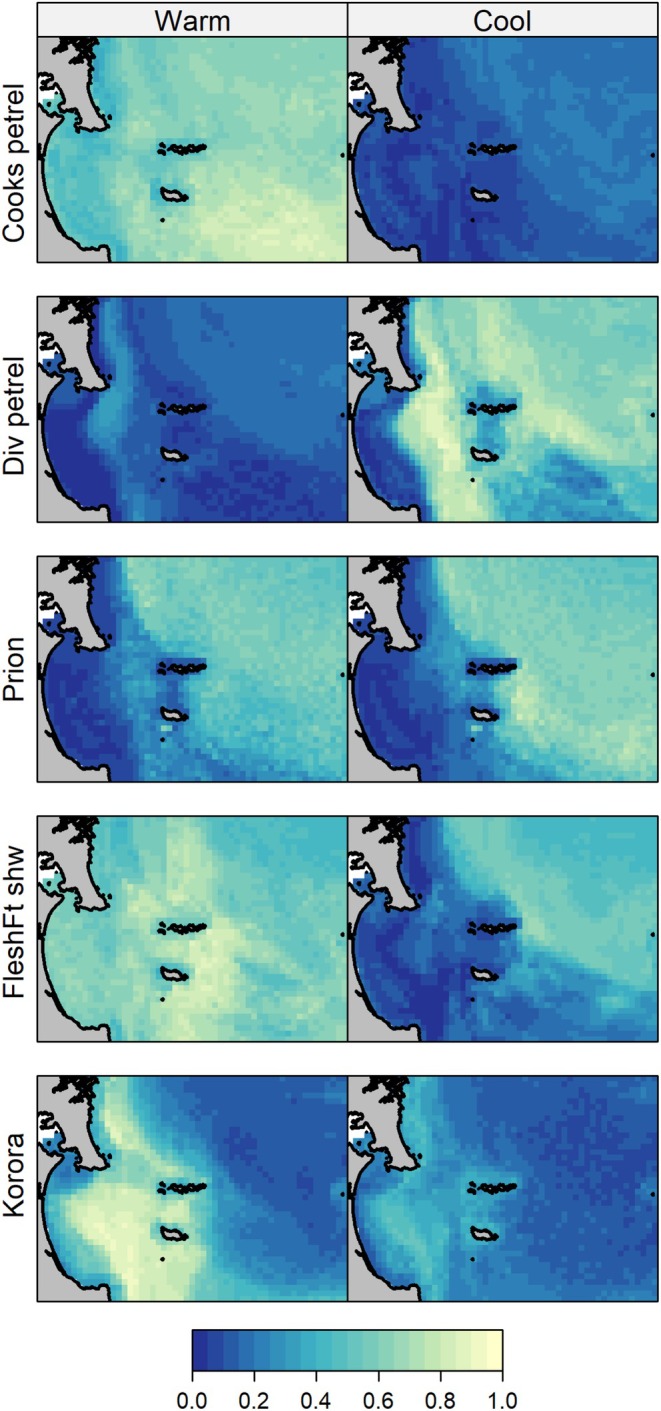
Seasonal predictions of probability of occurrence from species distribution models for five seabirds: Cooks/Pycroft petrel (Cooks petrel), diving petrel (Div petrel), prions spp. (Prion), flesh‐footed shearwater (FleshFt shw) and kororā/little penguin (Korora) for warm (December–March) and cool (June–September) conditions.

Of the nine seabird species with more than 20 unique occurrences (i.e., occurred in more than 20 seabird counts), robust species distribution models were able to be fitted for five species (Figure 4). These species showed distinct spatial and temporal patterns of distribution throughout the study area. The probability of occurrence of kororā/little penguin was predicted to be higher in the warm season, with hotspots throughout Te Ākau/Bream Bay and Te Paepae o Tu/Bream Tail, off Ocean Beach and between the Marotiri and Taranga Islands (Figure 4). In the cool season, the areas of highest relative importance were similar but suggested increased use of nearshore habitat around Te Whara/Whangārei Heads. Tītī/Cook's (and Pycroft) petrel was predicted to be widely distributed throughout the study area during summer, with hotspots in distribution in the south‐west, offshore of Taranga Island and with lower probability of occurrence inshore (Figure 4). Probability of occurrence during the cooler months was uniformly low. In contrast, probability of occurrence for kuaka/diving petrel was significantly lower during the warmer months. During the cool season, hotspots in distribution for diving petrel were notable in outer Te Ākau/Bream Bay in a band between the coastline and the Islands and east of Taranga Island. Toanui/flesh‐footed shearwater had a high probability of occurrence across both seasons, but with distinct seasonal distribution patterns including preference for offshore waters during the cool season. Tītī wainui/prions had a higher predicted probability of occurrence in offshore waters in both seasons, with higher values during the cool season.

A range of environmental variables was important for predicting the distribution of megafauna species using RFs (Table 3). Both static and dynamic variables made contributions to each species, with patterns in Sea Surface Temperature (SST) underpinning the key changes between seasons (Figure A2: Appendix S1). SST was ranked as the most important variable for all but two species, making over 15% contribution to the RF prediction for most models. Additionally, the horizontal gradient in SST was important for oceanic terehu/bottlenose dolphins, aihe/common dolphins, mautai/false killer whales, and tītī wainui/prions. Mixed layer depth (MLD) was an important predictor for aihe/common dolphins, tohorā/Bryde's whales, and kuaka/diving petrels, and slope was the second‐most important predictor for coastal terehu/bottlenose dolphins. A range of variables made moderate contributions to the kororā/little penguin predictions including chlorophyll *a* concentration, bathymetry, and turbidity (BBP). These variables, along with light irradiance at the seafloor (EBED) also made moderate contributions to models for kuaka/diving petrel, tītī wainui/prions, and toanui/flesh‐footed shearwater (Table 3).

**TABLE 3:** The standardised relative importance (% contribution) of environmental variables to the species distribution models for each marine mammal species.

Species	Bathy	BBP	BPI_fine	CHL	EBED	MLD	Season	Slope	SST	SSTGrad	TC
Terehu/Coastal bottlenose dolphin	5.01	8.14	4.71	11.87	6.07	5.36	4.09	13.68	30.55	5.72	4.79
Terehu/Oceanic bottlenose dolphin	6.51	6.36	5.21	4.74	6.89	6.72	6.50	4.96	24.53	21.31	6.26
Tohorā/Bryde's whale	8.36	8.17	8.46	7.08	10.68	12.92	2.48	6.96	19.85	9.02	6.03
Aihe/Common dolphin	9.85	8.24	8.56	9.02	7.01	15.24	1.22	7.45	15.05	11.86	6.49
Mautai/False killer whale	6.34	6.02	5.56	4.74	7.00	6.94	6.61	5.65	24.23	20.60	6.30
Kororā/Little penguin	11.12	9.32	6.07	14.01	8.08	6.88	3.71	5.81	19.64	8.4	6.98
tītī/Cook's/Pycroft petrel	7.29	5.92	5.98	10.57	7.32	5.41	8.05	6.71	27.74	8.29	6.73
Kuaka/diving petrel	8.23	9.15	8.45	8.05	8.59	14.96	1.34	8.19	19.47	6.84	6.71
tītī wainui/Prion spp.	7.95	18.03	12.19	8.11	15.07	8.30	0.40	5.63	5.57	13.16	5.58
Toanui/Flesh‐footed shearwater	92	9.50	8.96	8.79	9.32	7.65	4.66	7.86	17.70	9.13	7.51

*Note:* Colour shading indicates a gradient from the most important variable (yellow) to least important (blue). The environmental variables are; seafloor depth (Bathy), particulate backscatter (i.e., turbidity, BBP), bathymetric position index (BPI_fine), chlorophyll a concentration (CHL), light irradiance at the seafloor (EBED), season (categorical), mixed layer depth (MLD), slope of the seafloor (Slope), sea surface temperature (SST), horizontal gradient in SST (SSTGrad) and tidal current velocity (TC).

### 3.6 Acoustics

A total of 125 ten‐minute acoustic recordings were made and analysed for the detection of odontocete whistles. The proportion of recordings with positive detections was highest during autumn (64% of recordings), followed by winter (47%), summer (33%) and spring (37%) (Table A5: Appendix S1). Similarly, the mean number of positive detections per recording was highest in autumn and winter. ROCCA classification was unable to meaningfully detect differences between the two most common odontocetes (aihe/common and terehu/bottlenose dolphins), which is unsurprising given the generic whistle parameters of these species.

**FIGURE 5:**
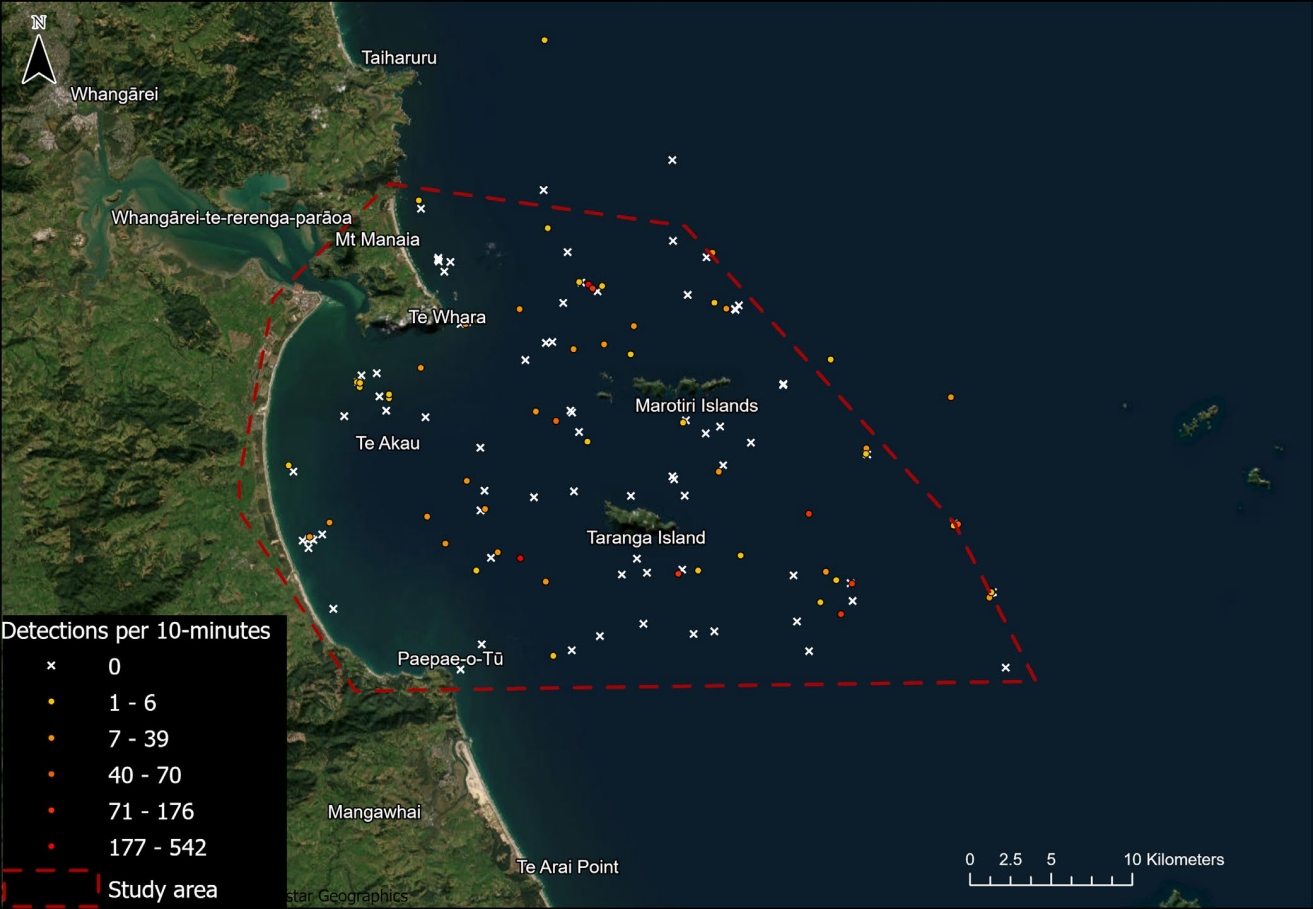
The location of acoustic recordings, the incidence of positive detections of odontocete whistles and the number of detections per 10‐min recording. Recordings with no detections are indicated as crosses.

Positive detections of odontocete whistles were made throughout the study area, although there was a high incidence of positive detections north of the Marotiri Islands and east of Taranga Island (Figure 5). Clusters of recordings with positive detections also occurred within Te Ākau/Bream Bay, particularly off the harbour entrance (Figure 5). Areas with scarce positive detections were between the two island groups and south of Taranga Island. For the former, it should be noted that the detection radius may be limited due to reduced propagation in more shallow water and screening from the islands themselves.

#### 3.6.1 Weaving Across Knowledge Systems

Key information on the marine megafauna of Te Ākau/Bream Bay was generated using both knowledge systems that can be summarised across seven key themes: species occurrence, spatial distribution, temporal patterns, behaviour, ecology, population dynamics and other (Table 4 and previous sections). When compared, information sourced from *mātauranga Māori* and conventional science reveals key insights for each knowledge theme (Table 4). Knowledge on species occurrence in the study area was greatly enhanced by integrating historical information from *mātauranga Māori* with systematic data on the diverse present‐day species assemblage. There was alignment in information on the spatial distribution of several species where predictions from SDMs were confirmed by knowledge on broad habitat use patterns (e.g., common dolphins). Both knowledge systems confirmed the study area has increased importance for several species during the summer months—adding additional evidence to this finding. Similarly, present‐day observations of foraging behaviour for many species were also reflected in *pūrakau* (legends) around foraging of whales during summer, substantiating the importance of the area as foraging habitat. Qualitative information on ‘high’ abundance of dolphin species aligned well with our identification of a comparatively large local population of terehu/bottlenose dolphin, reflecting that the area may have supported abundant dolphin populations over time. Pooled information on the ecology of the area provided a holistic understanding of ‘why’ the area may be important for marine megafauna. Lastly, incorporating information on the historical relationship between marine mammals and Patuharakeke helped to re‐establish this connection in the present day, providing a baseline for future *kaitiakitanga*/guardianship (Table 4).

**TABLE 4:** Summary of key insights from matāuranga Māori and conventional science for seven key knowledge themes relating to marine megafauna of Te Akau/Bream Bay.

Knowledge theme	Mātauranga Māori	Conventional science	Integrated insights
Species occurrence 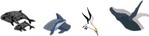	Rich information on marine mammal occurrence (place names/legends) Information on historical occurrences	High number of species detected Greater insights on non‐mammal megafauna (sharks/rays, seabirds)	Thorough baseline on diverse megafauna species Historical and present‐day occurrence = more robust baseline and opportunities for restoration
Spatial distribution 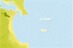	Broad areas of importance noted (e.g., offshore habitats/throughout the study area) Key habitats for some noted species	Predicted distribution for 10 spp. throughout the study area	Confirmation of important areas for several species with both knowledge systems = increased evidence Inference that key habitats have remained important over time
Temporal patterns 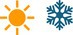	Summertime aggregations of whales Wintering ground for tohorā/southern right whales	Distinct occurrence patterns for marine mammals across seasons Variable cool/warm season distribution patterns for all species	Increased evidence for importance of study area during summer
Behavioural 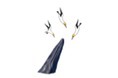	Foraging of whales common Historical nursery ground	Foraging behaviour regularly observed by all megafauna species	Study area has historically, and likely continues to be, important foraging habitat for multiple species groups
Population dynamics 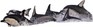	Qualitative historical ‘high’ abundance of delphinids	High abundance of terehu/bottlenose dolphins (150 identified individuals)	Study area likely supports dolphin populations with high local abundance, both historically and in the present day Suggestions of decline in abundance for some species
Ecology 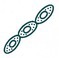	Mechanisms for the importance of the area (i.e., productivity) Relationships among species (mammals – seabirds) Connectivity between land and sea	Species – environment relationships (from SDMs)	Holistic understanding of the importance of the study area Connectivity between ecosystems
Other 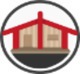	Historical relationships between people and biodiversity Capacity building opportunities in ‘alternative ways of knowing’	Capacity building opportunities in science and technology	Meaningful opportunities for reconnection between people and biodiversity, and between people from different backgrounds

*Note:* The benefits of integrating both knowledge systems are also provided as ‘integrated insights’ for each knowledge theme.

## 4 Discussion

### 4.1 Te Ākau/Bream Bay—A Hotspot for Marine Megafauna Considering Dual Knowledge Systems

This study has provided the first dedicated systematic survey of marine megafauna in the wider Te Ākau/Bream Bay area and combined this information with a stocktake of Māori knowledge on these taxa. Across both knowledge systems, our findings indicate the area provides critical habitat for marine megafauna, including a range of threatened and at‐risk species (Baker et al. 2019; Robertson et al. 2021). Few coastal areas in New Zealand hold such high diversity of marine mammals, sharks/rays and seabirds—with the nearby Hauraki Gulf being one of the few comparable locations (Dwyer et al. 2016; Gaskin and Rayner 2017; Stephenson, Hamilton, et al. 2023).

Pooled information across the two knowledge systems typically resulted in one of three situations for a given component of biodiversity: (1) alignment of knowledge, (2) complementarity of knowledge and (3) lack of alignment. Instances of alignment included the identification of the most common marine mammal species in the study area, confirmation of multiple ‘ecotypes’ for terehu/bottlenose dolphins, the importance of the area for foraging behaviour, seasonal patterns in marine mammal occurrence (e.g., greater occurrence in warmer months) and the colocation of some areas of importance for some species including tohorā/Bryde's whale and aihe/common dolphins (Table 4). For the latter, visual sightings (and subsequent model predictions Figure 3), acoustic detections (Figure 5) and Māori knowledge (Figure 2) all confirmed the importance of offshore habitat. As the mechanisms behind the generation of this information are so different, such specific instances of alignment across the knowledge systems add additional confirmation of these aspects of the area's biodiversity and are a key benefit of combining insights from multiple ‘ways of knowing’ (Gagnon and Berteaux 2009; Thornton and Scheer 2012; Teixeira et al. 2013; Alexander et al. 2019).

Due to the significant difference in temporal scales over which knowledge is generated via mātauranga Māori (centuries) and conventional research (2 years in this study) the two knowledge systems provided complementary insights on species occurrence (Table 4). For example, knowledge on the historical use of the harbour and surrounding waters by tohorā/southern right whales (e.g., legend on the relationship between Kauri and Tohorā) could not have been generated using conventional surveys (Figure 2). Tohorā/southern right whales have been extirpated from previously important wintering grounds in coastal NZ due to industrial whaling (Carroll et al. 2014), and so knowledge on key locations that could support recovery is highly valuable. Similarly, the historical use of the area as a corridor for migrating humpback whales was confirmed during *wānanga*, but not recorded during conventional surveys, likely due to the low (but recovering) abundance of this Oceania humpback population following illegal Soviet‐era whaling (Ivashchenko and Clapham 2014). The ability to incorporate historical information was also found by other studies that incorporate IK in biodiversity assessments and is seen as a substantial benefit (Thornton and Scheer 2012; Breton‐Honeyman et al. 2016; Marsh et al. 2022). The systematic sampling of modern‐day species occurrence and distribution via our conventional surveys provided robust information on the present‐day areas and seasons of importance for over ten megafauna species and allowed the quantification of uncertainty. This knowledge is important for informing *kaitiakitanga* (guardianship) and such explicit information was not readily available via Māori knowledge for all species, highlighting the benefits of considering both knowledge systems (Table 4).

Additional complementarity between the knowledge systems was evident, where Māori knowledge provided insights into the Māori understanding of the area's importance and the ecology of the area (Table 4). For example, several of the major geographic features are named for megafauna species and the name of the harbour specifically refers to a high abundance (i.e., gathering) of whales. *Pūrākau* (legends) that relate to the high productivity and abundance of the area's marine biota provide context for the diversity and abundance of megafauna encountered during our surveys. The high use of the area by wide‐ranging species (e.g., mautai/false killer whales, ūpokohue/pilot whales, tohorā/Bryde's whale) suggests there are persistent ecological features that may enhance prey availability and draw these species to the study area (Johnston et al. 2005; Hazen et al. 2011; Brough et al. 2023). This may occur via aggregation (e.g., entrainment) or via increases in productivity via upwelling events or oceanographic features such as fronts, eddies or island wakes (Owen 1981; Johnston et al. 2005; Johnston and Read 2007), that are known to occur along this coast (Stevens et al. 2021). The ability for Māori knowledge to provide a holistic understanding of ‘why’ this area may be important for the marine megafauna further showcases the value of considering dual knowledge systems for baseline biodiversity studies, a finding that has been shown in similar studies elsewhere (Stern and Humphries 2022; Gryba et al. 2025).

*Kōrero tuku iho* (oral traditions) around the naming of Whangarei Terenga Parāoa (Whangarei Harbour; Figure 2) was one of the few instances where there was a lack of alignment between Māori and conventional scientific knowledge. Some narratives spoke of the importance of the harbour for Parāoa/sperm whale, yet the species was not recorded during our surveys and, based on our scientific understanding of the species' ecology, we would not expect sperm whales in this shallow, coastal habitat. Additionally, we recorded a much broader diversity of megafauna species during our vessel‐based surveys than knowledge holders spoke about during *wānanga*. This was especially true for more transient and pelagic species: mautai/false killer whales, blue whale, manta ray, most petrels and shearwaters. The lack of alignment between IK and contemporary science systems may reflect a lack of context around language (e.g., *Parāoa* may have historically referred to whales more generally), different units and scales of observation and quantitative vs. qualitative frameworks (Alexander et al. 2019; Stern and Humphries 2022; Berkes 2009; Hikuroa 2017), or due to a loss of knowledge on certain aspects of biodiversity (Teixeira et al. 2013; Hikuroa 2017; Ataria et al. 2018). The latter consideration may be pertinent to the case of more transient, pelagic species in this study, where opportunities to experience the more offshore parts of the study area are not often forthcoming for Patuharakeke members due to socioeconomic conditions. That this study delivered a ‘reconnection’ between Patuharakeke members and these species through our vessel‐based *wānanga* was an important outcome that will help to rebuild local knowledge of these important species (Table 4).

### 4.2 Key Species of Te Ākau

In NZ, tohorā/Bryde's whales are listed as Nationally Critical, the highest designation in the NZ Threat Level Classification system (Baker et al. 2019). Critical habitat for the species is mostly known from the Hauraki Gulf, where ship‐strike impacts on the local population have been successfully managed for several decades (Constantine et al. 2015). Sighting rates of Bryde's whales reported within the Hauraki Gulf are similar or lower than those generated in Te Ākau/Bream Bay by this study (Dwyer et al. 2016; Hamilton et al. 2023) using similar approaches to generate SPUE (albeit with different platforms) and mātauranga Māori confirmed the regular use of the study area by this species (Figure 2). Thus, it is reasonable to conclude that Bryde's whales have at least similar (and potentially higher) relative abundance in Te Ākau/Bream Bay compared to the currently recognised New Zealand hotspot for the species. Use of coastal embayments by tohorā/Bryde's whales is well known in the inner Hauraki Gulf (Wiseman et al. 2011; Stephenson, Hamilton, et al. 2023) and the Bay of Islands (Baker and Madon 2007), and the high use of such habitats in this study raises concerns for the impact of stressors associated with coastal habitat.

Our findings suggest that this area is regularly used by both ecotypes of terehu/bottlenose dolphins. The coastal ecotype is currently classed as Nationally Endangered (Baker et al. 2019). The outputs from the SDMs, distribution of acoustic detections and Māori knowledge on the occurrence of the species show that coastal terehu/bottlenose dolphins use large parts of the study area throughout most of the year (Figures 2, 3 and 5). Sighting rates during systematic surveys (mean = 0.011 km^−1^) were similar to or greater than those reported from other areas of known importance for the species, e.g., Bay of Islands (0.007 km^−1^; Tezanos Pinto 2009), the inner Hauraki Gulf (< 0.003 km^−1^; Dwyer et al. 2016) and Queen Charlotte & Pelorus Sound (mean = 0.005 km^−1^, Merriman et al. 2009). Photo‐ID of nearly 150 individual coastal bottlenose with recapture rates above 70% is suggestive of a large local population with a high degree of site fidelity to this area. All local scale (e.g., < 100 km) areas in NZ with local populations of coastal terehu/bottlenose typically have fewer than 150 identifiable extant individuals (Bennington et al. 2020; Brough et al. 2025) and thus our work suggests one of NZ's largest local populations may reside in Te Akau/Bream Bay.

Along with oceanic terehu/bottlenose, mautai/false killer whales frequent the study area regularly between December and April. They are typically considered an understudied offshore species (R. Baird 2018). The waters off north‐eastern New Zealand are one of the few documented regions globally where mautai/false killer whales enter continental shelf waters for prolonged periods of time (Zaeschmar et al. 2014). Within this region, the study area is of particular importance during autumn months as evidenced by long‐term sightings data (Zaeschmar et al. 2020) and the sightings generated as part of this study. The persistent use of coastal habitat by mautai/false killer whales is unique among sub‐tropical/temperate regions (Zaeschmar et al. 2014).

Aihe/common dolphins were the most frequently sighted cetacean species during systematic surveys and were confirmed as the most common species during *wānanga*. While we could not reliably classify odontocete whistles to species, aihe/common dolphins were likely the species most frequently detected during our acoustic recordings. These findings are consistent with their widespread abundance in NZ waters (Stockin and Orams 2009). The sighting rates of common dolphins reported in this study (mean = 0.020 km^−1^) are similar to those reported in the Hauraki Gulf by (Stockin et al. 2008; mean = 0.021 km^−1^)^1^ and by Dwyer et al. (2016) (range ~0.008–0.042 km^−1^). Seasonal fluctuations in sighting rates were also similar between this study and (Stockin et al. 2008), with higher sighting rates in winter. The more offshore distribution of common dolphins matches closely with that observed in the Bay of Islands (Constantine and Baker 1997), the Bay of Plenty (Meissner et al. 2015), and in the Hauraki Gulf (Dwyer et al. 2020; Stephenson, Hamilton, et al. 2023) and also matches insights from Māori knowledge (Figure 2). However, common dolphins also regularly occur within inshore habitat during the Hauraki Gulf (Stockin et al. 2008; Dwyer et al. 2020; Stephenson, Hamilton, et al. 2023).

The number of seabirds (24 species) recorded in our study is comparable to locations with known high diversity and suggests Te Ākau/Bream Bay is an important location for seabirds. However, it should be noted that our species list includes several species complexes that are likely representative of multiple species (e.g., prions, skuas, storm‐petrels). Further, common nearshore species (e.g., shags, some terns) are not well represented likely due to a lack of survey effort in nearshore and harbour habitat. Twenty‐seven species of seabirds are known to breed within the Hauraki Gulf and surrounding areas, and the area has a high diversity of transient species (Gaskin and Rayner 2017; Gaskin 2021). Surveys off the coast of the Far North district have reported a similar number of species to this study (23 species) (Winterle Daudt 2024). Recent surveys of coastal habitat in several locations on the east coast of the South Island reported 27 seabird species in Dunedin, 13 at Moeraki, 12 at Timaru, 29 at Banks Peninsula (Bourke and Bennington 2024). Several threatened species were observed during this study (NZ storm petrel, brown skua, and black petrel) along with a range of species classified as ‘at‐risk’. The common occurrence of several threatened or at‐risk species substantiates the importance of this area for seabirds.

Most megafauna species detected during our surveys had strong seasonal patterns in occurrence and distribution (Figures 3 and 4), although many could be found throughout the study area and across seasons (e.g., Tables 1 and A5: Appendix S1). Seasonal variation in distribution is well known for many species throughout NZ waters (Brough et al. 2019; Bennington et al. 2023; Stephenson, Hamilton, et al. 2023). The seasonality in the distribution of marine mammals is typically thought to follow that of their prey (Torres et al. 2008; Brough et al. 2023), or to be related to habitat requirements for seasonal life history processes (e.g., calving) (Rayment et al. 2015; Sprogis et al. 2018). The seasonality in species occurrence and distribution aligned closely with *mātauranga* (Māori knowledge) associated with the name of Whangārei Terenga Parāoa—where oral tradition suggests the name originates from the gathering of whales during the warmer months for feeding. Feeding aggregations/behaviour were regularly observed by all whale species detected during our surveys during summer.

Highly dynamic patterns in seabird distribution were also noted in this study, particularly the lower occurrence of most species during the cool season (Figure 4). While many species breed in the surrounding area, several undergo seasonal migrations to areas throughout the Pacific during the NZ winter, and thus the lower occurrence during the cool season is expected (Heather and Robertson 2015). We also encountered lower cool season occurrence for species expected to be present year‐round including kororā/little penguin, tākapu/Australasian Gannet and pakahā/fluttering shearwater (Figure 4). For these resident species, it may be that the study area is not as routinely used during the cooler season, and individuals forage more selectively elsewhere. Alternatively, the survey conditions experienced during the cooler season may reduce the sightability of seabirds in general, causing some downward bias on sighting rates and probability of occurrence (Lambert et al. 2024).

Spatially, each of the common seabird species had distinct distribution patterns. Kororā/little penguin are central place foragers and their foraging (at‐sea) distribution is influenced by the proximity of their colonies which occur on both offshore island groups and around Te Whara/Whangārei Heads. Tītī/Cook's and Pycroft petrel were distributed widely throughout the study area during the warm season, but had hotspots around Taranga and towards Hauturu—breeding colonies for both species (Taylor 2013; Taylor and Rayner 2022). During the cool season, the key habitat for kuaka/diving petrels overlapped with that of tohorā/Bryde's whales, an interspecific association also confirmed during *wānanga*. Several species (e.g., Toanui/flesh‐footed shearwaters, kororā/little penguin) exhibited seasonal inshore‐offshore patterns of space use that are common in seabirds and typically relate to the seasonal availability of their prey community (Montevecchi et al. 2009; Suryan et al. 2016).

It should be noted that some of the seasonal patterns in occurrence identified in this study may be subject to bias associated with reduced survey effort during the winter (June) and spring (September). These seasons had lower effort (Table A2: Appendix S1) and the data used to characterise species occurrence was generated over a single survey for each season, compared with Summer and Autumn seasons, that had at least 2 full surveys. While the full study area was surveyed during winter and spring, we cannot rule out that temporally dynamic processes may have impacted sighting rates during these seasons. Thus, seasonal occurrence patterns during these months should be considered a ‘snapshot’ given the environmental conditions at the time of survey, noting that this is a common approach when undertaking initial biodiversity assessments for cetaceans (Weir et al. 2007; Melly et al. 2018; Dolman et al. 2014; Stephenson, Hamilton, et al. 2023; Putra et al. 2025). Additionally, we cannot account for the likelihood that the inter‐annual variability in distribution and occurrence may have influenced our analyses and thus further work should repeat surveys across multiple years to determine the long‐term consistency of the patterns observed in this study.

### 4.3 Kaitiakitanga (Guardianship)

*Kaitiakitanga* is a Māori concept of guardianship/management, that is a key role of local *iwi/hapū* (tribes/sub‐tribes) towards their local environment and that may be enacted via various legislative and non‐legislative processes. As a coastal *hapū*, Patuharakeke are strongly invested in maintaining this role for their future generations and in recognition of the significant inter‐relationships between the health of their people and the health of the ocean. Proposed increases in shipping and port development may present risks to these waters. Mortality of tohorā/Bryde's whales from ship‐strike has had population‐level consequences in the Hauraki Gulf (Constantine et al. 2015) and has been observed elsewhere (Laist et al. 2001; Van Der Hoop et al. 2013). The study area is not currently protected by restrictions on commercial shipping that limit ship‐strike impact in the Hauraki Gulf. As both *mātauranga Māori* and conventional surveys suggest Te Ākau/Bream Bay is an important habitat for tohorā/Bryde's whales, and the area faces proposed increases to commercial shipping and port development, such protection should be considered. Increases in shipping, and the noise/light pollution it produces, may also pose risks to the other megafauna species recorded during this study (Marley et al. 2017; Wisniewska et al. 2018; Ryan et al. 2021).

Adjacent land use from coastal development may have impacts on species that regularly use near‐shore habitat (terehu/coastal bottlenose, tohorā/Bryde's whale, maki/killer whales, kororā/little penguins, pakahā/fluttering and rako/Buller's shearwaters) via effects on coastal water quality (e.g., turbidity, nutrient enrichment) and sedimentation may degrade foraging habitat or impact prey availability/catchability (Jefferson et al. 2009; Culloch et al. 2016; Hawkins et al. 2017). Insights from *wānanga* highlight the strong relationship between some species (e.g., tohorā/southern right whales) and land and reflect the vulnerability of coastal species to land use impacts. There are currently proposals for seabed mining and an expansion of port infrastructure and shipping in this area. These proposals should carefully consider the likelihood of negative adverse impacts given the importance of this area for megafauna and should be carefully managed to avoid overlap with areas of importance for the diverse species documented here. The impacts of seafloor mining on marine megafauna are poorly understood, but impacts associated with noise pollution are likely (Todd et al. 2015; Thompson et al. 2023). A range of other stressors including bycatch from commercial fishing, exploitation of prey stocks, climate change, disease and combinations of several stressors, may impact populations in this important area. Ongoing research should aim to assess the population status of these *taonga* (treasured) species and discern the impact of any stressors that occur within this area.

## 5 Conclusions

Using systematic surveys, opportunistic encounters, and pooling of insights from Māori knowledge, this study has generated a robust baseline on the ecology of marine megafauna within Te Ākau/Bream Bay. Accurate documentation on the diversity, occurrence, and distribution of key species generated from conventional science and a *te aō Māori* (*Māori* worldview) approach provides Patuharakeke and their wider partners with robust information that can be used to inform the practice of *kaitiakitanga* in this *rohe moana* (tribal waters) of special significance. This study has also demonstrated the significant value of weaving IK within baseline assessments for marine megafauna by showcasing the alignment of the two approaches and the ability of Māori knowledge to highlight historical species occurrence and abundance. Using both knowledge systems is clearly advantageous and is highly recommended for ongoing research and management of marine megafauna and their habitats.

## Author Contributions

**Tom Brough:** conceptualization (equal), data curation (equal), formal analysis (lead), funding acquisition (equal), investigation (equal), methodology (equal), project administration (equal), writing – original draft (lead), writing – review and editing (lead). **Hollie Kereopa:** formal analysis (equal), investigation (equal), methodology (equal), writing – original draft (equal), writing – review and editing (equal). **Taryn Shirkey:** conceptualization (equal), formal analysis (equal), funding acquisition (equal), investigation (equal), methodology (equal), writing – review and editing (equal). **Jochen Zaeschmar:** conceptualization (equal), data curation (equal), formal analysis (equal), funding acquisition (equal), investigation (equal), methodology (equal), resources (lead), writing – original draft (equal), writing – review and editing (equal). **Eva Leunissen:** data curation (equal), formal analysis (equal), writing – original draft (equal), writing – review and editing (equal). **Dave Milner:** conceptualization (equal), supervision (equal), writing – original draft (equal), writing – review and editing (equal). **Juliane Chetham:** conceptualization (equal), funding acquisition (supporting), methodology (equal), supervision (lead), writing – original draft (equal), writing – review and editing (equal).

## Funding

This work was supported by New Zealand Ministry of Business Innovation and Employment ‐ Vision Matauranga Capability Fund (C01X2120).

## Disclosure

Positionality Statement: Four of the authors (HK, TS, DM, JC) of this study are Māori and are members of the hapū (subtribe) Patuharakeke, while holding wider tribal affiliations (e.g., Ngatiwai). The remaining authors (TB, JZ, EL) are tangata tiriti (NZ European), with longstanding connections to the waters of Te Tai Tokerau (the Far North of NZ). This work was commissioned to meet the aspirations of Patuharakeke with respect to their desire to reconnect with the marine megafauna of the rohe moana (tribal waters), reignite mātauranga Māori on these species and generate information to inform kaitiakitanga of their rohe moana. All aspects of the project were fully co‐developed and delivered under a partnership between Patuharakeke and the science team. A range of te reo Māori terms are used throughout the text; please see Appendix 2: Appendix S1 for a glossary of terms.

## Conflicts of Interest

The authors declare no conflicts of interest.

## Supporting information


**Appendix S1:** ece372558‐sup‐0001‐AppendixS1.docx.

## Data Availability

All data from conventional scientific surveys collected and analysed as part of this study are available upon request via the Zenodo data portal at https://doi.org/10.5281/zenodo.17873774.

## Endnotes

^1^
Adjusted from sightings per 60 min using vessel speed provided (15 km).
