# Supplementary material for: Tere Tohorā, Karanga Tāngata: Weaving Māori Knowledge With Conventional Science to Characterise a Biodiversity Hotspot for Marine Megafauna in an Area Facing Multiple Anthropogenic Impacts
Source: Ecol Evol. 2025 Dec 15;15(12):e72558. doi: 10.1002/ece3.72558 (PMC12705289; doi:10.1002/ece3.72558)
Supplement: Supplementary file 1 — Appendix S1: ece372558‐sup‐0001‐AppendixS1.docx. [file ECE3-15-e72558-s001.docx]

# Appendix 1

*Table A1. The environmental variables used for species distribution modelling of marine mammals and seabirds in this study.*

| **Variable** | **Name** | **Description** | **Spatial resolution (m)** | **Temporal resolution** | **Reference** |
| --- | --- | --- | --- | --- | --- |
| Bathy | Bathymetry | Depth of the seafloor | 500 | Static | National scale dataset; NIWA unpublished, updated in 2020 |
| BBP | Particulate backscatter | The particulate backscatter coefficient at 555 nm (m^-1^), which is highly correlated with turbidity measurements by optical backscatter sensors. | 500 | Monthly | NIWA-SCENZ; Pinkerton et al. 2022 |
| BPI_fine | Bathymetric position index (fine-scale) | Bathymetric position index (BPI) is a measure of where a referenced location is relative to the locations surrounding it. Terrain metrics were calculated using an inner annulus of 12 km and a radius of 62 km. | 500 | Static | National scale dataset; NIWA unpublished, updated in 2020 |
| CHL | Chlorophyll-a concentration | A proxy for the biomass of phytoplankton present in the surface ocean (to ~30 m depth) | 500 | Monthly | NIWA-SCENZ; Pinkerton et al. 2022 |
| EBED | Seabed incident irradiance | Broadband (400–700 nm) incident irradiance (E m-2 d ^-1^) at the seabed, averaged over a whole year | 500 | Monthly | NIWA-SCENZ; Pinkerton et al. 2022 |
| MLD | Mixed layer depth | The depth that separates the homogenised mixed water above from the denser stratified water below | 500 | Static | National scale dataset; NIWA unpublished, updated in 2020 |
| Slope | Slope | Bathymetric slope was calculated from water depth and is the degree change from one depth value to the next | 500 | Static | National scale dataset; NIWA unpublished, updated in 2020 |
| SST | Sea surface temperature | Blended from OI-SST (Reynolds et al. 2002) ocean product and MODISAqua SST coastal product. Long term (2002 – 2021) average values at 250 m resolution | 500 | Monthly | NIWA-SCENZ; Pinkerton et al. 2022 |
| SSTGrad | Sea surface temperature gradient | Smoothed magnitude of the spatial gradient of annual mean SST. This indicates locations in which frontal mixing of different water bodies is occurring (Leathwick et al. 2006). | 500 | Monthly | This study |
| TC | Tidal Current speed | Maximum depth-averaged (New Zealand bathymetry) flows from tidal currents calculated from a tidal model for New Zealand waters (Walters et al. 2001) | 500 | Static | National scale dataset; NIWA unpublished, updated in 2020 |

*Table A2:*

| Survey | December 2022 | March 2023 | June 2023 | September 2023 | December 2023 | January 2024 | March 2024 |
| --- | --- | --- | --- | --- | --- | --- | --- |
| Dates | 5 Dec 2022 – 9 Dec 2022 | 13 Mar 2023 – 17 Mar 2023 | 2 June 2023 – 6 June 2023 | 24 Sept 2023 – 29 Sept 2023 | 3 Dec 2023 – 10 Dec 2023 | 15 Jan 2024 – 20 Jan 2024 | 18 Mar 2024 – 23 Mar 2024 |
| Season | Summer | Autumn | Winter | Spring | Summer | Summer | Autumn |
| Broad season | Warm | Warm | Cool | Cool | Warm | Warm | Warm |
| Kilometres on effort | 207 | 195 | 196 | 232 | 255 | 226 | 228 |

*Table A3: Seasonal sightings of marine megafauna made during systematic surveys (S) and opportunistic encounters (O). The total number of sightings and the total number of individuals sighted are provided along with the species’ mean sighting rate (n sightings/km effort) calculated using systematic sightings only. Little penguins are reported under megafauna as they were surveyed using the same methods.*

| **Season**  **Species** | **Spring** | | **Summer** | | **Autumn** | | **Winter** | | **Total sightings** | | **Total individuals** | | **sightings/km** |
| --- | --- | --- | --- | --- | --- | --- | --- | --- | --- | --- | --- | --- | --- |
|  | S | O | S | O | S | O | S | O | S | O | S | O | S |
| **Marine mammals** |  |  |  |  |  |  |  | - |  |  |  |  |  |
| Aihe/ common dolphin (*Delphinus delphis*) | 2 | - | 14 | 5 | 10 | 5 | 5 | 0 | 31 | 10 | 1377 | 315 | 0.020 |
| Terehu / coastal bottlenose dolphin (*Tursiops truncatus)* | - | - | 9 | 8 | 8 | 7 | - | 1 | 17 | 16 | 502 | 534 | 0.011 |
| Terehu / oceanic bottlenose dolphin (*Tursiops truncatus)* | - | - | 2 | 12 | 6 | 20 | - | - | 8 | 32 | 740 | 4550 | 0.005 |
| Maki / killer whale (*Orcinus orca)* | - | - | 1 | - | - | 1 | - | - | 1 | 1 | 5 | 8 | 0.001 |
| Mautai / false killer whale (*Pseudorca crassidens*) | - | - | 1 | 12 | 3 | 20 | - | - | 4 | 32 | 340 | 2560 | 0.003 |
| Upokohue / long-finned pilot whale (*Globicephala melas)* | - | - | - | 6 | - | 4 | - | - | - | 10 | - | 255 | - |
| Tohorā / Bryde’s whale *(Balaenoptera edeni brydei)* | 3 | - | 9 | 3 | 8 | 6 | - | - | 20 | 7 | 32 | 11 | 0.013 |
| Blue whale (*Balaenoptera musculus)* | 1 | - | - | - | - | - | - | - | 1 | - | 1 | - | 0.001 |
| Kekeno / New Zealand fur seal *(Arctocephalus forsteri)* | - | - | - | - | - | - | 2 | - | 2 | - | 2 | - | 0.001 |
| **Elasmobranchs** |  |  |  |  |  |  |  |  |  |  |  |  |  |
| Mangōpare / hammerhead shark (*Sphyrna zygaena)* | - | - | 11 | - | - | - | - | - | 11 | - | 11 | - | 0.007 |
| Mako shark *(Isurus oxyrinchus)* | - | - | 1 | - | - | - | - | - | 1 | - | 1 | - | 0.001 |
| Blue shark *(Prionace glauca)* | - | - | - | - | - | - | 1 | - | 1 | - | 1 | - | 0.001 |
| Manta ray (*Mobula birostris)* | - | - | 7 | 7 | 1 | - | - | - | 8 | 7 | 14 | 9 | 0.009 |
| **Penguins** |  |  |  |  |  |  |  |  |  |  |  |  |  |
| Kororā / little penguin (*Eudyptula minor*) | 4 | - | 41 | - | 52 | - | - | - | 104 | - | 209 | - | 0.136 |

*Table A4: A summary of the species recorded in all seabird counts across the seasonal surveys (months) of this study. The occurrence (percentage of all counts in a given month) and the mean number of individuals per count are given for all species observed throughout this study.*

|  | **December** | | **January** | | **March** | | **June** | | **September** | | **Total** |
| --- | --- | --- | --- | --- | --- | --- | --- | --- | --- | --- | --- |
|  | % counts | mean ind. | % counts | mean ind. | % counts | mean ind. | % counts | mean ind. | % counts | mean ind. | % counts |
| Arctic skua *(Stercorarius parasiticus)* | 0 | 0 | 0 | 0 | 4 | 1.0 | 0 | 0 | 0 | 0 | 0.4 |
| Tākapu / Australasian gannet *(Morus serrator)* | 30 | 2.9 | 25 | 1.4 | 28 | 2.0 | 29 | 1.9 | 42 | 1.9 | 29.6 |
| Takoketai / Black petrel *(Procellaria parkinsoni)* | 0 | 0 | 1 | 1.0 | 24 | 1.5 | 0 | 0 | 5 | 1.5 | 4.0 |
| Karoro / Black-backed gull *(Larus dominicanus)* | 5 | 1.0 | 1 | 1.0 | 0 | 0 | 6 | 1.0 | 18 | 2.7 | 5.4 |
| Toroa / Black-browed albatross *(Thalassarche melanophris)* | 2 | 1.0 | 0 | 0 | 0 | 0 | 0 | 0 | 0 | 0 | 0.4 |
| Toroa / Buller's albatross *(Thalassarche bulleri)* | 0 | 0 | 0 | 0 | 0 | 0 | 0 | 0 | 18 | 1.6 | 3.1 |
| Rako / Buller's shearwater *(Ardenna bulleri)* | 61 | 8.7 | 36 | 6.5 | 24 | 2.0 | 0 | 0 | 39 | 4.5 | 34.5 |
| Cape petrel *(Daption capense)* | 0 | 0 | 0 | 0 | 0 | 0 | 0 | 0 | 3 | 1.0 | 0.4 |
| Tītī / Cook’s & Pycroft petrel (*Pterodroma cookie/pycrofti)* | 18 | 3.5 | 38 | 4.5 | 0 | 0 | 0 | 0 | 0 | 0 | 17.5 |
| Kuaka / Northern diving petrel *(Pelecanoides urinatrix)* | 39 | 3.8 | 0 | 0 | 0 | 0 | 71 | 4.9 | 61 | 8.7 | 29.1 |
| Toanui / Flesh-footed shearwater *(Ardenna carneipes)* | 50 | 3.1 | 37 | 7.5 | 20 | 22.0 | 0 | 0 | 21 | 2.1 | 29.1 |
| Pakahā / Fluttering shearwater *(Puffinus gavia)* | 77 | 26.2 | 21 | 11.2 | 16 | 4.0 | 31 | 2.8 | 42 | 3.6 | 36.8 |
| Ōi / Grey-faced petrel *(Pterodroma gouldi)* | 0 | 0 | 0 | 0 | 4 | 1.0 | 0 | 0 | 0 | 0 | 0.9 |
| Toroa / Grey headed albatross *(Thalassarche chrysostoma)* | 0 | 0 | 0 | 0 | 0 | 0 | 0 | 0 | 5 | 1.0 | 0.4 |
| Totorore / Little shearwater *(Puffinus assimilis)* | 0 | 0 | 1 | 1.0 | 0 | 0 | 3 | 2.0 | 0 | 0 | 0.9 |
| Northern giant petrel *(Macronectes halli)* | 2 | 1.0 | 0 | 0 | 0 | 0 | 0 | 0 | 11 | 1.3 | 2.2 |
| Takahikare-raro / NZ storm petrel *(Fregetta maoriana)* | 2 | 1.0 | 4 | 1.3 | 0 | 0 | 0 | 0 | 3 | 1.0 | 2.2 |
| Tītī wainui / Prion spp. (*Pachyptila spp.)* | 18 | 18.5 | 7 | 2.0 | 0 | 0 | 0 | 0 | 24 | 31.0 | 10.3 |
| Takapunga / Red-billed gull (*Chroicocephalus novaehollandiae scopulinus)* | 5 | 3.5 | 0 | 0 | 12 | 2.3 | 9 | 5.3 | 8 | 2.7 | 4.9 |
| Skua spp. (*Stercorarius spp.)* | 2 | 4.0 | 1 | 1.0 | 0 | 0 | 0 | 0 | 0 | 0 | 0.9 |
| Storm petrel spp. (*Fregetta spp.)* | 5 | 15.5 | 0 | 0 | 0 | 0 | 3 | 2.0 | 3 | 2.0 | 1.8 |
| Takahikare-moana / White-faced storm petrel (*Pelagodroma marina maoriana)* | 27 | 3.4 | 15 | 1.7 | 4 | 1.0 | 0 | 0 | 32 | 2.9 | 16.6 |
| Tara / White-fronted tern *(Sterna striata)* | 2 | 3.0 | 1 | 3.0 | 0 | 0 | 3 | 2.0 | 0 | 0 | 1.3 |

*Table A5: Table of acoustic recordings by season, proportion of recordings with positive odontocete detections, and mean number of detections per recording.*

| **Season** | **Number of recordings** | **Proportion of recordings with +ve detections** | **Mean detections per recording** |
| --- | --- | --- | --- |
| Summer | 52 | 33% | 15.5 |
| Autumn | 39 | 64% | 47.4 |
| Winter | 15 | 47% | 41.9 |
| Spring | 19 | 37% | 19.1 |


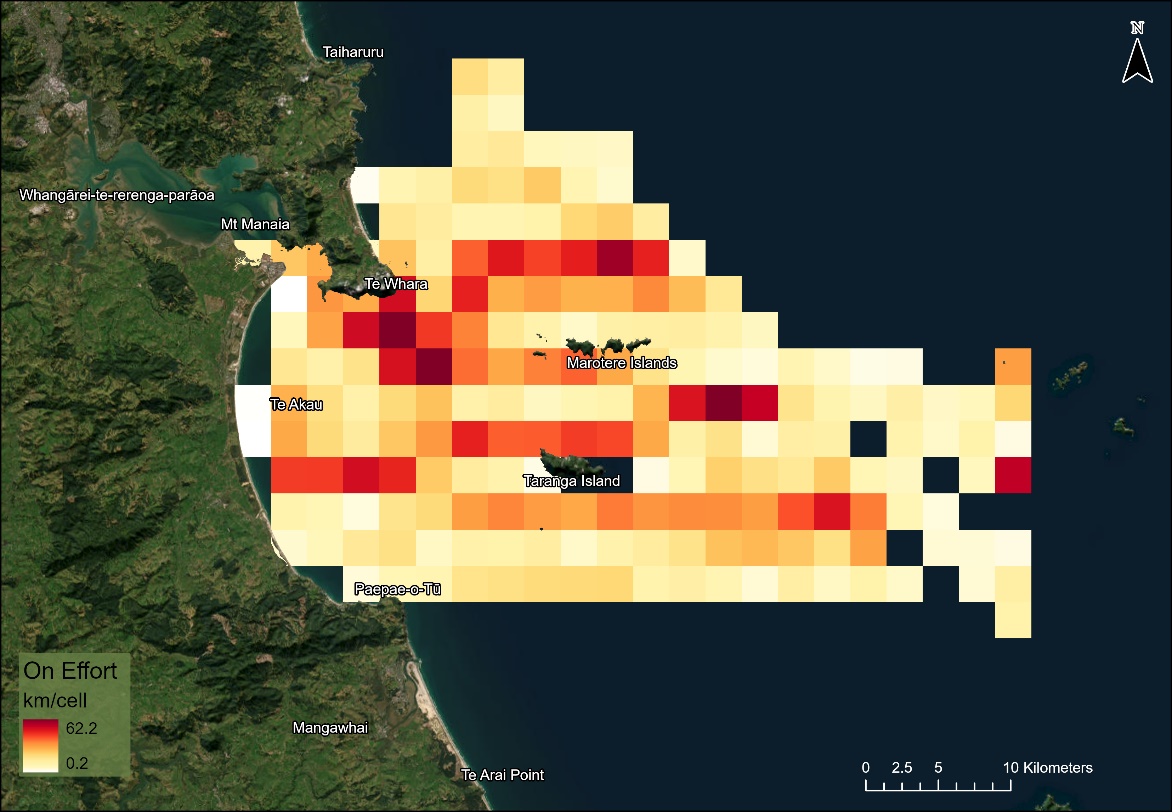


Figure A1: The distribution of 'on-effort' survey effort from systematic vessel-based surveys for marine megafauna in Te Akau/Bream Bay


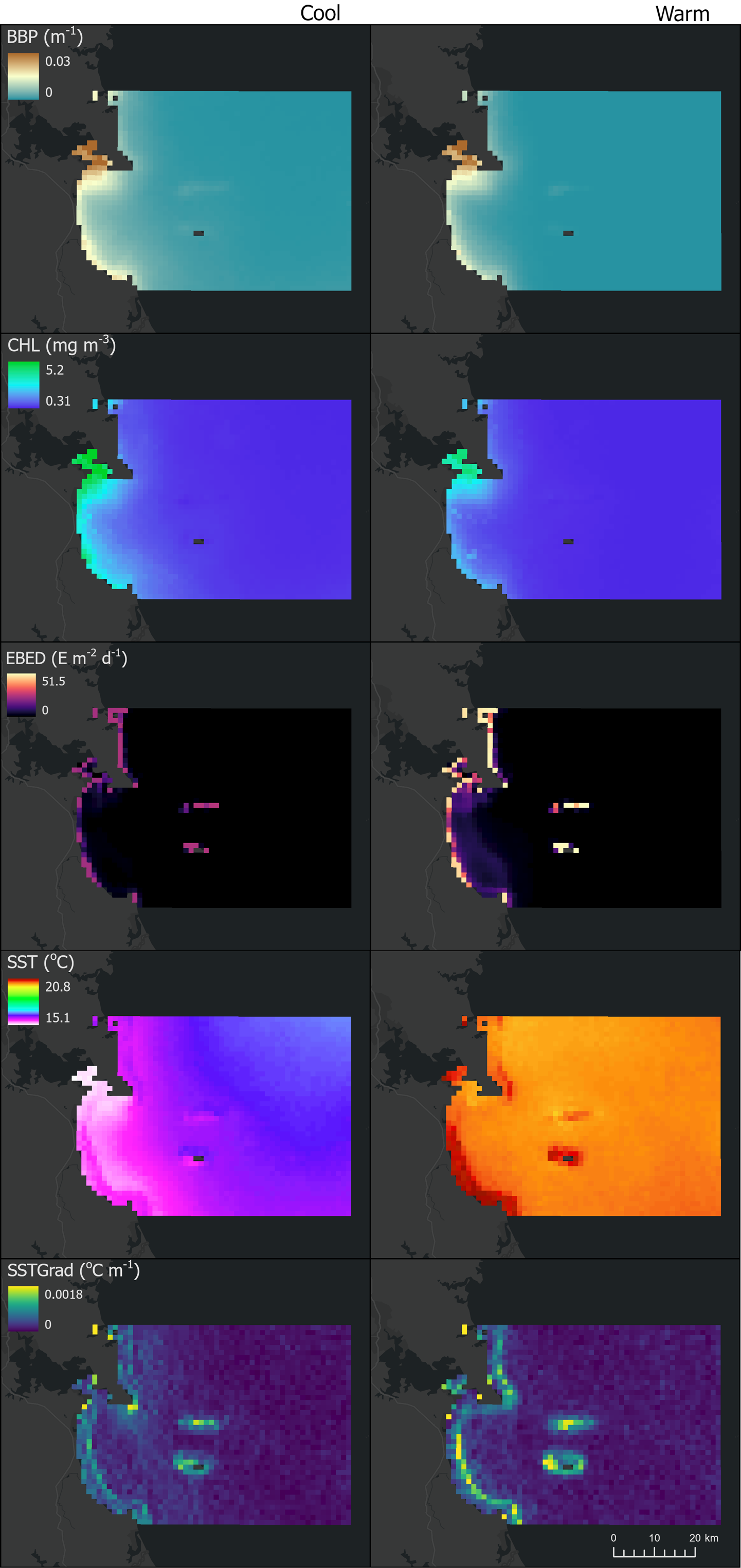


*Figure A2 Maps of the dynamic variables used in the SDMs, for the cool season (left column) and warm season (right column)*

# Appendix 2

## Glossary

**Te reo Māori terms used in text**

Awa River

Hapū Māori sub-tribe

Hauhake Traditional harvesting

Hononga Partnership

Iwi Māori tribe

Kaitiakitanga Guardianship

Kaumatua Elders

Kōrero tuku iho Oral traditions

Mana Respect/strength

Marae Meeting house

Mātauranga Māori Traditional Māori knowledge

Moana Ocean

Ngātiwai Iwi (tribe) along the north-east coast of NZ

Pūrākau Legends

Rohe moana Tribal waters

Taonga Treasure/treasured

Te aō Māori The Māori worldview/world

Tikanga Cultural protocols

Tohunga Experts

Tuakana Kin

Tupuna Ancestor

Tupuna taonga Treasured ancestor

Whakapapa Genealogy/genealogical relationships

Whānau Family/extended family

Whānui Wider community

Whenua Land

**Common species names**

Aihe Short-beaked common dolphins (*Delphinus delphis*)

Kuaka Northern diving petrel (*Pelecanoides urinatrix*)

Maki Killer whale (*Orcinus orca)*

Mangōpare Hammerhead shark (*Sphyrna zygaena*)

Mautai False killer whale (*Pseudorca crassidens*)

Ōi Grey-faced petrel (*Pterodroma gouldi*)

Paikea Humpback whales (*Megaptera novaeangliae*)

Pakahā Fluttering shearwater (*Puffinus gavia*)

Rako Buller’s shearwater (*Ardenna bulleri*)

Takahikare White-faced storm petrel (*Pelagodroma marina maoriana*)

Tākapu Australasian gannet (*Morus serrator*)

Takoketai Black petrel (*Procellaria parkinsoni*)

Terehu Common bottlenose dolphin (*Tursiops truncatus)*

Tītī Cook’s and Pycroft petrel (*Pterodroma spp.)*

Toanui Flesh-footed shearwater (*Ardenna carneipes*)

Tohorā Whales (generally) but also refers specifically to southern right whale

Toroa Black-browed albatross (*Thalassarche melanophris*)

Ūpokohue Long-finned pilot whales (*Globicephala melas*)

**Place names**

Aorangi and Tawhiti Rahi The Poor Knights Islands

Aotea Great Barrier Island

Hauturu Little Barrier Island

Marotiri “The Chicks” Island group

Motu Kino and Pokohinu Mokohinau Islands

Taranga “The Hen” Island

Te Ākau Bream Bay

Te Whara Whangārei Heads

Te-ika-a-Māui North Island of New Zealand

Tūturu Sail Rock

Whangārei Terenga Parāoa Whangārei Harbour

# Appendix 3

The settings used for PAMGuard’s Whistle and Moan detector were: Minimum frequency=3kHz; Maximum frequency = 37kHz; Connection type = 8 sides/diagonals; Minimum length= 10 slices; Minimum total size = 50 pixels; Crossing/joining = Relink; Maximum cross length = 5 slices; Median filter length = 61; Subtraction constant = 0.02; Smoothing: ON; Threshold: 5 dB), and a detection threshold of 5 dB.

The output from this detector was then passed to PAMGuard’s ROCCA classifier to identify potential species (Oswald et al. 2007) using the ‘Temperate Pacific’ classifier model (Oswald et al. 2015). This process generated an output table of ROCCA statistics for all contours detected (n = 1,144,481). To reduce the number of false positive detections, any detections classified as “Ambiguous” were excluded (69%). Contours with a duration less than 0.2 s were also excluded (a further 27%), which was based on manual inspection of false positive contours. The data were then summarised per recording to generate the number of detections per recording and were matched to recording location and time.

To verify the accuracy of the detection process, 15 recordings (12% of full set) were randomly selected for manual checking, with a random selection from each season. Each of these 15 recordings was checked by listening to the entire 10-minute recording, noting whether any whistles or moans were heard. If vocalisations were detected, the recording was considered a positive detection. This was compared to the automatic detection using PAMGuard for those recordings to assess rates of false positive and false negative detections generated by the automatic classifier.
